# Five New Biphenanthrenes from *Cremastra appendiculata*

**DOI:** 10.3390/molecules21081089

**Published:** 2016-08-19

**Authors:** Liang Liu, Jun Li, Ke-Wu Zeng, Yong Jiang, Peng-Fei Tu

**Affiliations:** 1 State Key Laboratory of Natural and Biomimetic Drugs, School of Pharmaceutical Sciences, Peking University Health Science Center, Beijing 100191, China; enjoyyz@163.com (L.L.); ZKW@bjmu.edu.cn (K.-W.Z.); yongjiang@bjmu.edu.cn (Y.J.); 2 Medical College, Yangzhou University, Yangzhou 225009, China; 3 Jiangsu Key Laboratory of Zoonosis, Jiangsu Co-innovation Center for Prevention and Control of Important Animal Infectious Diseases and Zoonoses, Yangzhou University, Yangzhou 225009, China; 4 Modern Research Center for Traditional Chinese Medicine, Beijing University of Chinese Medicine, Beijing 100029, China; drlj666@163.com

**Keywords:** *Cremastra appendiculata*, biphenanthrene, cytotoxic activity

## Abstract

Five new biphenanthrenes, cremaphenanthrenes A–E (**1**–**5**), along with six known ones, were isolated from the ethanolic extract of the tubers of *Cremastra appendiculata* (D. Don) Makino (Orchidaceae). Their structures were elucidated on the basis of extensive spectroscopic analyses. All the compounds obtained were tested in vitro for cytotoxic activities against colon (HCT-116), cervix (Hela), and breast (MDA-MB-231) cancer cell lines. They all showed moderate or weak cytotoxicities to the above cancer cell lines.

## 1. Introduction

Phenanthrenes are a rather uncommon class of aromatic metabolites, which are presumably formed by oxidative coupling of the aromatic rings of stilbene precursors, and have been reported in higher plants, mainly in the Orchidaceae family [1]. The phenanthrenes isolated thus far may be classified into three major groups: monophenanthrenes, biphenanthrenes, and triphenanthrenes, and most natural phenanthrenes occur in monomeric form [1]. To date, less than one hundred biphenanthrenes and two triphenanthrenes have been isolated from the Orchidaceae family [2,3,4,5,6,7,8,9,10,11,12,13]. Biphenanthrenes have been reported to possess various biological activities including cytotoxicity, antimicrobial, spasmolytic, anti-inflammatory, antiplatelet aggregation, and antiallergic activities [1]. Currently, the cytotoxic activities of biphenanthrenes have attracted much interest, and biphenanthrenes may potentially be served as novel class of antitumor candidate [4].

**Figure 1:**
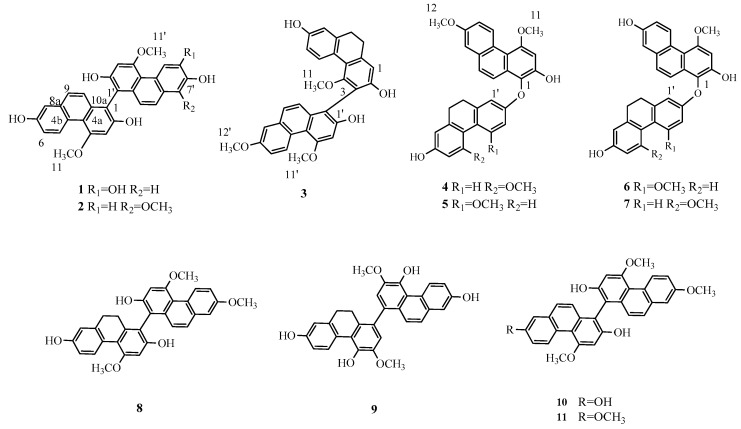
Structures of compounds **1**–**11** isolated from *C. appendiculata.*

The tuber of *Cremastra appendiculata* (D. Don) Makino (Orchidaceae) is one main source of “Shancigu”, which is a famous traditional Chinese medicine with a long history for treating cancers [14]. About forty phenanthrenes, including ten biphenanthrenes, have been isolated from this title plant in previous phytochemical investigations [2,3,13,15,16]. The in vitro cytotoxic activities of some biphenanthrenes are better than the corresponding monomeric phenanthrenes [1,2]. In a previous article, eight new benzylphenanthrenes, and a few known compounds, were reported from this herb [13,15,16]. Our continuing investigations on the constituents of this plant have led to the isolation of five new biphenanthrenes, together with six known ones (Figure 1). In this paper, we report the structural identification of five previously unreported biphenanthrenes, namely cremaphenanthrenes A–E (**1**–**5**), as well as their cytotoxic activities.

## 2. Results and Discussion

The tubers of *C. appendiculata* were extracted with 95% EtOH to yield a dark brown residue, which was suspended in distilled water and partitioned successively with petroleum ether (PE), ethyl acetate (EtOAc), and *n*-butyl alcohol (*n*-BuOH). The PE and EtOAc partitions were chromatographed over silica gel, Sephadex LH-20 and ODS columns, and semi-preparative HPLC to obtain eleven biphenanthrenes (**1**–**11**) (Figure 1). Their structures were elucidated based on extensive spectroscopic analyses (Supplementary Materials).

### 2.1. Structure Elucidation

**Figure 2:**
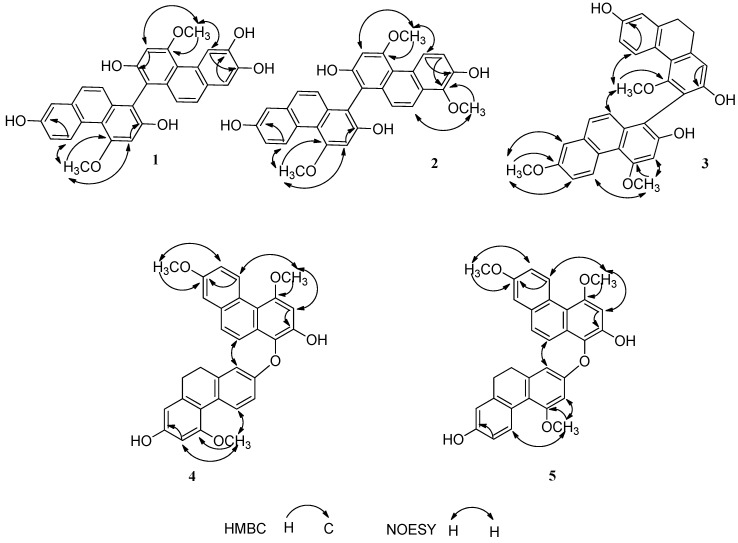
Key NOESY and HMBC correlations of compounds **1**–**5**.

Compound **1** (Table 1 and Table 2, Figure 2) was obtained as a brown amorphous powder. The positive ion HR-ESI-MS showed an ion at *m*/*z* 1011.2639 [2M + Na]^+^ and established the molecular formula as C_30_H_22_O_7_. The IR spectrum showed absorption bands at 3239, 1612, 1588, and 1206 cm^−1^ ascribable to hydroxyl and aromatic functions, respectively. The UV spectrum showed absorption maximum at 203, 264, and 308 nm. The ^1^H-NMR spectrum displayed eleven aromatic protons signals including one set of ABX coupling systems at δ_H_ 9.36 (1H, d, *J* = 9.0 Hz, H-5), 7.09 (1H, dd, *J* = 9.0, 3.0 Hz, H-6) and 7.04 (1H, d, *J* = 3.0 Hz, H-8); four singlets signals at δ_H_ 6.97 (1H, s, H-3), 6.92 (1H, s, H-3′), 8.96 (1H, s, H-5′) and 7.02 (1H, s, H-8′); two pairs of doublets signals at δ_H_ 7.28 (1H, d, *J* = 9.0 Hz, H-9), 6.90 (1H, d, *J* = 9.0 Hz, H-10), 7.20 (1H, d, *J* = 9.0 Hz, H-9′ ), and 6.71 (1H, d, *J* = 9.0 Hz, H-10′). In addition, two methoxy singlets signals at δ_H_ 4.10 (6H, s, H-11, H-11′) were observed as well. The ^13^C-NMR spectrum of **1** displayed twenty eight aromatic carbons (including seven oxygenated quaternary aromatic ones, whose chemical shifts were above 140 ppm) and two methoxy carbons signals. These data, especially the presence of the deshielded protons signals at δ_H_ 9.36 (H-5) and 8.96 (H-5′) indicated compound **1** was an asymmetrical biphenanthrene with five hydroxyls and two methoxyls as substituents. The substituent positions of **1** were further confirmed by 2D-NMR experiments. Based on the HMBC correlations from H-3 to C-1, C-2, C-4 and C-4a, H-5 to C-4a, C-6, C-7 and C-8a, H-6 to C-4b and C-8, H-8 to C-4b, C-6, C-7 and C-9, H-9 to C-4b, C-8, C-8a and C-10a, H-10 to C-1, C-4a and C-8a, H-11 to C-4, and NOESY correlations from δ_H_ 4.10 (H-11) to 6.97 s (H-3) and 9.36 (H-5), one phenanthrene unit was determined to be 2,7-hydroxy-4-methoxyphenanthrene. HMBC correlations from H-3′ to C-1′, C-2′, C-4′ and C-4a′, H-5′ to C-4a′, C-7′ and C-8a′, H-8′ to C-4b′, C-6′, C-7′ and C-9′, H-9′ to C-4b′, C-8′, C-8a′ and C-10a′, H-10′ to C-1′, C-4a′ and C-8a′, H-11′ to C-4′, and NOESY correlations from δ_H_ 4.10 (H-11′) to 6.92 (H-3′) and 8.96 (H-5′) revealed the other phenanthrene unit was 2′,6′,7′-trihydroxy-4′-methoxyphenanthrene. According to the HSQC and HMBC spectra, δ_C_ 111.3 (C-1) and δ_C_ 110.6 (C-1′) were two quaternary aromatic carbons, suggesting the two phenanthrene units to be connected directly by C-1 and C-1′. Therefore, the structure of **1** was established as 2,7,2′,6′,7′-pentahydroxy-4,4′-dimethoxy-1,1′-biphenanthrene, and named as cremaphenanthrene A.

Compound **2** (Table 1 and Table 2, Figure 2) was isolated as a brown amorphous powder. The molecular formula of **2** was determined as C_31_H_24_O_7_ from the negative ion HR-ESI-MS at *m*/*z* 507.1455 [M − H]^−^. The IR and UV spectra were similar to those of **1**. The molecular weight of **2** was fourteen mass units more than that of **1**. Moreover, two typical deshielded protons signals at δ_H_ 9.38 (H-5) and 9.17 (H-5′) in ^1^H-NMR spectrum, twenty eight aromatic carbons (including seven oxygenated quaternary aromatic ones), three methoxy carbons signals appeared in ^13^C-NMR spectrum, which suggested that **2** was also an asymmetrical biphenanthrene with four hydroxyls and three methoxyls as substituents. The substitution patterns of **2** were similar to those of **1** except for a methoxyl located at C-8′ and the absence of a hydroxyl assigned to C-6′, respectively. This was further confirmed by the HMBC correlations from H-8′-OCH_3_ to C-8′, H-6′ to C-8′, as well as the NOESY correlation from H-8′-OCH_3_ to H-9′. Thus, the structure of **2** was elucidated as 2,7,2′,7′-tetrahydroxy-4,4′,8′-trimethoxy-1,1′-biphenanthrene, and named as cremaphenanthrene B.

Compound **3** (Table 1 and Table 2, Figure 2) was obtained as a brown amorphous powder. A positive [M + H]^+^ ion at *m*/*z* 495.1795 was found in the HR-ESI-MS spectrum of **3**, and given the molecular formula as C_31_H_26_O_6_. The 1D NMR spectra of **3** displayed two typical pairs of protons signals at δ_H_ 2.73–2.78 (4H, m, H-9,10), twenty six aromatic (including six oxygenated quaternary aromatic carbons), three methoxy carbons, and two methylenes signals, which suggested that compound **3** was an asymmetrical phenanthrene-9,10-dihydrophenanthrene dimer with three hydroxyls and three methoxyls as substituents. The substitution patterns of **3** were determined by comprehensive analyses of HSQC, HMBC, and NOESY spectra, especially the correlations from H-11 (δ_H_ 3.14) to H-5 (δ_H_ 8.08); H-11′ (δ_H_ 4.15) to H-3′ (δ_H_ 6.96) and H-5′ (δ_H_ 9.50); H-12′ (δ_H_ 3.92) to H-6′ (δ_H_ 7.17) and H-8′ (δ_H_ 7.25) in the NOESY spectrum. The linkage between the two moieties was deduced to be C-3 (δc 117.7) and C-1′ (δc 110.9) by quaternary aromatic carbon nature, chemical shifts, and the molecular composition, especially by the NOESY correlation from H-11 (δ_H_ 3.14) to H-10′ (δ_H_ 7.36). Subsequently, the structure of **3** was determined as 2,7,2′-trihydroxy-4,4′,7′-trimethoxy-9,10-dihydro-3,1′-biphenanthrene, and named as cremaphenanthrene C. 

Compound **4** (Table 3 and Table 4, Figure 2) was obtained as a brown amorphous powder. The molecular formula was assigned as C_31_H_26_O_6_ on the basis of a negative [M − H]^−^ ion at *m*/*z* 493.1637 in HR-ESI-MS of **4**. In the ^1^H-NMR spectrum, eleven aromatic protons signals, three methoxy singlet signals, and two typical pairs of protons signals at δ_H_ 2.61–2.63 (4H, m, H-9′, H-10′) assigned to 9′,10′-dihydrophenanthrene were observed. In the ^13^C-NMR spectrum, twenty six aromatic and two methoxy carbons signals were displayed. These data indicated the presence of biphenanthrene, which was made up of a phenanthrene moiety and a 9′,10′-dihydrophenanthrene moiety. In the HMBC spectrum, correlations from H-3 to C-1, C-2, C-4 and C-4a, H-5 to C-4a, C-7 and C-8a, H-6 to C-4b, H-8 to C-4b, C-6 and C-9, H-9 to C-4b, C-8 and C-10a, H-10 to C-1 and C-4a, H-11 to C-4, and H-12 to C-7, together with the NOESY correlations from H-11 (δ_H_ 4.12) to H-3 (δ_H_ 6.94) and H-5 (δ_H_ 9.45), H-12 (δ_H_ 3.91) to H-6 (δ_H_ 7.20) and H-8 (δ_H_ 7.26) were observed, which revealed the phenanthrene moiety to be 2-hydroxy-4,7-dimethoxyphenanthrene. The HMBC correlations from H-1′ to C-2′ and C-4a′, H-4′ to C-10a′, C-2′ and C-4b′, H-6′ to C-4b′, C-7′ and C-8′, H-9′ to C-4b′, C-10a′ and C-8′, H-10′ to C-4a′, C-1′ and C-8a′, H-11′ to C-5′, along with the NOESY correlations from H-11′ (δ_H_ 3.80) to H-4′ (δ_H_ 8.05) and H-6′ (δ_H_ 6.38) were observed, which suggested the presence of 2′,7′-dihydroxy-5′-methoxy-9,10-dihydrophenanthrene. C-1 (δ_C_ 130.9) of phenanthrene moiety was shifted downfield, and it was a quaternary aromatic carbon, so it might be linked with oxygen, which indicated the 9,10-dihydrophenanthrene moiety and the phenanthrene moiety were connected through C-1 and C-2′ or C-1 and C-7′. The NOESY correlation from H-10 (δ_H_ 7.70) to H-1′ (δ_H_ 6.67) confirmed the existence of C-1 and C-2′ linkage patterns. Thus, the structure of **4** was finally established as 2,7′-dihydroxy-4,7,5′-trimethoxy-9′,10′-dihydro-1,2′-biphenanthreneether, and named as cremaphenanthrene D.

Compound **5** (Table 3 and Table 4, Figure 2) was obtained as a brown amorphous powder. The molecular formula was deduced as C_31_H_26_O_6_ according to the appearance of a negative [M − H]^−^ ion at *m*/*z* 493.1665 in the HR-ESI-MS of **5**. Compounds **5** and **4** have identical molecular formula and similar IR, UV, and 1D-NMR spectra, as well as the same connection site between phenanthrene moiety and 2′,7′-dihydroxy-4′-methoxy-9,10-dihydrophenanthrene. This was confirmed by the vital NOESY correlations from H-10 (δ_H_ 7.74) to H-1′ (δ_H_ 6.20). Thus, we elucidated the structure of **5** as 2,7′-dihydroxy-4,7,4′-trimethoxy-9′,10′-dihydro-1,2′-biphenanthreneether, and named as cremaphenanthrene E. 

Compounds **6** and **7** (Table 3 and Table 4, Figure 2) were obtained as brown amorphous powders. The molecular formula was deduced as C_30_H_24_O_6_ according to their HR-ESI-MS. Their IR, UV, and 1D-NMR spectra were similar with those of **4** and **5**, but their molecular weights were fourteen mass units less than those of **4** and **5**. All of the above information indicated the character of biphenanthreneether of **6** and **7**. Compared with **5**, **6** had a hydroxyl located at C-7 instead of a methoxyl in the same position of **5**, which was confirmed by the HMBC correlations from H-5 to C-7. Thus, the structure of **6** was elucidated as 2,7,7′-trihydroxy-4,4′-dimethoxy-9′,10′-dihydro-1,2′-biphenanthreneether. It was a known compound with the name blestrin C [12]. In comparation with **4**, **7** also possessed a hydroxyl located at C-7 instead of a methoxyl in the corresponding position of **4**, the HMBC correlations from H-5 to C-7 further confirmed the deduction. Therefore, the structure of **7** was defined as 2,7,7′-trihydroxy-4,5′-dimethoxy-9′,10′-dihydro-1,2′-biphenanthreneether, which was a known compound having the name blestrin D [12]. The assignments of ^1^H- , ^13^C-NMR data of blestrin C (**6**) and D (**7**) in the literature were partly different with ours [12]. In this paper, we assigned 1D NMR data of the two compounds based on the comprehensive analyses of ^1^H-NMR, ^13^C-NMR, HSQC, HMBC, and NOESY spectra.

Other four known biphenanthrenes were identified as 4,7, 4′-trimethoxy-9′,10′-dihydro(1,1′-biphenanthrene)-2,2′,7′-triol (**8**) [11], phochinenin B (**9**) [6], 2,7,2′-trihydroxy-4,4′,7′-trimethoxy-1,1′-biphenanthrene (**10**) [2], and 2,2′-dihydroxy-4,4′,7,7′-tetramethoxy-1,1′-biphenanthrene(**11**) [2] by comparison spectroscopic data with those in literatures.

### 2.2. Cytotoxicity Assay

The cytotoxicities of **1**–**11** were evaluated by the MTT method [2], using paclitaxel as a positive control. Their cytotoxicities against human colon (HCT-116), cervix (Hela), and breast (MDA-MB-231) cancer cell lines were determined. The results (Table 5) indicated that **1**–**11** showed moderate or weak cytotoxicities to the tested cancer cell lines. Among them, compounds **10** and **11** showed moderate cytotoxicities to all the above cancer cell lines with IC_50_ values range of (12.13 ± 0.38)–(17.43 ± 3.07) μmol/L. They are all hexasubstituted phenanthrene-phenanthrene dimer with hydroxyl and methoxyl located at C-2, C-2′, C-4, C-4′, C-7, and C-7′. Compound **8** was a phenanthrene-9,10-dihydrophenanthrene dimer with the identical substitution pattern to **10**. However, **8** only had weak cytotoxicities against the tested three cancer cell lines. Compounds **3** and **8** had the same phenanthrene and 9,10-dihydrophenanthrene moieties with the different linkage positions (1-1′ or 3-1′ connections, respectively), while the cytotoxicities of **3** were better than those of **8**, which indicated that the linkage position of phenanthrene and 9,10-dihydrophenanthrene moieties affected the activity. All of the above results suggested that the cytotoxicities of biphenanthrenes might be associated with the following factors: the number of substituted hydroxyl and methoxyl, the hydroxyl and methoxyl location, the linkage position of two phenanthrene moieties, and the type of moiety (phenanthrene or 9,10-dihydrophenanthrene).

## 3. Experimental Section

### 3.1. General Experimental Procedures

UV spectra were run on a Shimadzu UV-2450 spectrometer (Shimadzu, Tokyo, Japan). IR spectra were recorded on a Thermo Nicolet NEXUS-470 FTIR spectrometer (Thermo-Niolet, Madison, WI, USA). HR-ESI-MS were determined by a Bruker APEX IV FT-MS (7.0 T) (Bruker, Bremen, Germany) and a Waters Xevo G2 Q-TOF/YCA mass spectrometers (Waters, Milford, MA, USA). NMR spectra were recorded on a Varian Inova-500 (Varian, Palo Alto, CA, USA) and a Bruker Avance-600 FT NMR spectrometers (25 °C) (Bruker, Karlsruhe, Germany). Semi-preparative HPLC were run on a Dionex Ultimate 3000 instrument (Dionex, Sunnyvale, CA, USA) equipped with 170U UV Detector (254 nm) and an ODS column (Waters Co., Milford, MA, USA; 250 mm × 10 mm, 5 μm). Column chromatography (CC) was performed using silica gel (200–300 mesh, Qingdao Marine Chemistry Ltd., Qingdao, China), Sephadex LH-20 (Amersham Biosciences, Uppsala, Sweden) and ODS C_18_ (40–63 μm, Merck, Darmstadt, Germany). TLC was carried out on glass precoated silica gel (GF254) plates (Qingdao Marine Chemistry Ltd., Qingdao, China). Spots were visualized under UV light and detected by spraying with 10% H_2_SO_4_ in EtOH followed by heating. All purified compounds submitted for bioassay were at least 95% pure, as judged by HPLC analyses.

### 3.2. Plant Material

The tubers of *C. appendiculata* were collected in Yunnan province in June 2011. The plant materials were identified by one of the authors (Prof. P.F. Tu), and a voucher specimen (No. DJL20110628) was deposited at the Herbarium of Peking University Modern Research Center for Traditional Chinese Medicine.

### 3.3. Extraction and Isolation

The powdered, dry tubers of *C. appendiculata* (30 kg) were extracted with 95% EtOH (100 L) three times, each for two hours. After filtration and evaporation, a dark brown residue was obtained. The residue was suspended in distilled water and partitioned successively with PE (3 × 10 L), EtOAc (3 × 10 L), and *n*-BuOH (3 × 15 L). Part of the PE fraction (33.3 g) was separated by silica gel column chromatography (CC, 6 × 60 cm) eluted with a gradient of PE–EtOAc (50:1–0:100) to give nine fractions (Fraction A–I) based on TLC analysis. Fraction H (1.4 g) was chromatographed on a silica gel column (3 × 30 cm) using a gradient of PE–acetone (4:1–0:100) to get nine fractions (H1–H9). Fraction H8 (58.0 mg) was then subjected to CC (2.5 × 30 cm) on a silica gel employing CHCl_3_-acetone (10:1) to yield compound **11** (6.0 mg). Fraction I (0.9 g) was separated by silica gel CC (2.5 × 30 cm) eluted with a PE–acetone gradient (4:1–0:100) to give six fractions (Fraction I1–I6). Fraction I6 (140.0 mg) was then subjected to CC (3.0 × 80 cm) on Sephadex LH-20 using CHCl_3_:MeOH (2:1) as eluent to obtain compound **10** (10.2 mg). The EtOAc fraction was isolated by silica gel CC (8.0 × 60 cm) eluted with a gradient of CHCl_3_:MeOH (50:1–0:100) to give nine fractions (Fraction A–I) using TLC analysis. Thirteen fractions (A1–A13) were obtained from Fraction A (21.6 g) chromatographed on a silica gel column (5.0 × 50 cm) using a gradient of PE:acetone (5:2–0:100) as eluent. Fraction A9 (1.8 g) was subjected to CC (3.0 × 80 cm) on Sephadex LH-20 employing CHCl_3_:MeOH (2:1) to yield 10 fractions (A9-1 to A9-10). Fraction A9-9 (58.5 mg) was separated by semi-preparative HPLC, using acetonitrile (ACN):H_2_O (3:2, 2.0 mL/min), then MeOH:H_2_O (4:1, 2.0 mL/min) as mobile phase to yield compound **4** (2.6 mg, t_R_ = 31.5 min) and **5** (2.8 mg, t_R_ = 33 min)**.** Fraction A11 (2.1 g) was separated into nine fractions (A11-1 to A11-9) by a Sephadex LH-20 CC (3.0 × 80 cm) and washed with CHCl_3_:MeOH (1:2). Fraction A11-5 (135.8 mg) was applied to a silica gel CC (2.5 × 30 cm) using PE:CHCl_3_:MeOH (5:5:1), then further purified by semi-preparative HPLC, eluted with ACN:H_2_O (11:9, 2.0 mL/min) as the mobile phase, to yield compounds **8** (1.8 mg, t_R_ = 34.9 min) and **3** (1.6 mg, t_R_ = 28.9 min). Fraction A12 (2.7 g) was subjected by a silica gel CC (3.0 × 30 cm) and eluted with a gradient of CHCl_3_:EtOAc (3:1–0:100) to afford 10 fractions (Fraction A12-1 to A12-10). Fraction A12-5 (282.3 mg) was separated into 10 fractions (A12-5-1 to A12-5-10) by a Sephadex LH-20 CC (3.0 × 80 cm) using MeOH. Fraction A12-5-6 (48.3 mg) was purified by semi-preparative HPLC, using ACN-H_2_O (11:9, then 21:29, 2.0 mL/min) as the mobile phase, to yield compounds **6** (3.2 mg, t_R_ = 55.5 min) and **7** (2.6 mg, t_R_ = 61.0 min). Fraction A12-6 (372.6 mg) was separated on a RP-C18 silica gel CC (3.0 × 30 cm) eluted with a gradient of MeOH–H_2_O (2:1–100:0) to give 10 fractions (A12-6-1 to A12-6-10). Compound **2** (3.2 mg, t_R_ = 35.0 min) was finally obtained from Fraction A12-6-3 (45.2 mg) through semi-preparative HPLC (ACN:H_2_O (11:9, 2.0 mL/min)). Original extract Fraction B of EtOAc fraction (12.91 g) was separated on a silica gel CC (5.0 × 50 cm) using PE:acetone:MeOH (5:5:1.2-0:0:100) to obtain seven fractions (B1–B7). Fraction B2 (3.85 g) was then further fractionated on a Sephadex LH-20 CC (3.0 × 80 cm) eluted with CHCl_3_:MeOH (1:1) to give eight fractions (B2-1 to B2-8). Fraction B2-4 (540.0 mg) was separated on a silica gel CC (3.0 × 30 cm) employing CHCl_3_–acetone (5:1) to get three fractions (B2-4-1 to B2-4-3), Fraction B2-4-1 (135.2 mg) was purified by a RP-C18 CC (2.5 × 30 cm) eluted with a MeOH:H_2_O gradient (2:1–100:0), then by a Sephadex LH-20 CC (3.0 × 80 cm) and washed with MeOH to yield compound **9** (28.2 mg). Fraction D of EtOAc fraction (3.5 g) was separated into six fractions (D1–D6) by a silica gel CC (3.0 × 30 cm) eluted with a gradient of PE:EtOAc (1:1–0:100). Fraction D5 (260.0 mg) was further applied to a Sephadex LH-20 CC (3.0 × 80 cm) using a CHCl_3_:MeOH gradient (1:2–0:100) to afford four fractions (D5-1 to D5-4). Fraction D5-4 (20.0 mg) was purified by a RP-C18 silica gel CC (2.0 × 20 cm) washed with a gradient of MeOH:H_2_O (3:1–100:0) to yield two fractions (D5-4-1 to D5-4-2), compound **1** (5.2 mg, t_R_ = 35.0 min) was then purified from Fraction D5-4-2 (11.0 mg) by semi-preparative HPLC, using MeOH-H_2_O (3:2, 2.0 mL/min) as the mobile phase.

### 3.4. Spectroscopic Data

*Cremaphenanthrene A* (**1**): brown amorphous powder. UV λ_max_ (MeOH) nm (log ε): 203 (0.77), 264 (1.81), 308 (0.29). IR (KBr) ν_max_ (cm^−1^): 3239, 1612, 1588 and 1206. ^1^H- and ^13^C-NMR data: Table 1 and Table 2. Positive ion HR-ESI-MS *m*/*z* 1011.2639 [2M + Na]^+^ (calcd for C_30_H_22_O_7,_ 1011.2623).

*Cremaphenanthrene B* (**2**): brown amorphous powder. UV λ_max_ (MeOH) nm (log ε): 212 (0.50), 264 (1.12), 310 (0.22). IR (KBr) ν_max_ (cm^−1^): 3345, 1615, 1463, 1370 and 1023. ^1^H- and ^13^C-NMR data: Table 1 and Table 2. Negative ion HR-ESI-MS *m*/*z* 507.1455 [M − H]^−^ (calcd for C_31_H_24_O_7,_ 507.1449).

*Cremaphenanthrene C* (**3**): brown amorphous powder. UV λ_max_ (MeOH) nm (log ε): 211 (1.19), 263 (1.33). IR (KBr) ν_max_ (cm^−1^): 3402, 2931, 1611, 1586, 1460 and 1275. ^1^H- and ^13^C-NMR data: Table 1 and Table 2. Positive ion HR-ESI-MS *m*/*z* 495.1795 [M + H]^+^ (calcd for C_31_H_26_O_6__,_ 495.1808).

*Cremaphenanthrene D* (**4**): brown amorphous powder. UV λ_max_ (MeOH) nm (log ε): 211 (0.81), 263 (1.02). IR (KBr) ν_max_ (cm^−1^): 3416, 2933, 1611, 1357, 1215 and 1084. ^1^H- NMR and ^13^C-NMR data: Table 3 and Table 4. Negative ion HR-ESI-MS *m*/*z* 493.1637 [M − H]^−^ (calcd for C_31_H_26_O_6__,_ 493.1657).

*Cremaphenanthrene E* (**5**): brown amorphous powder. UV λ_max_ (MeOH) nm (log ε): 211 (0.63), 263 (0.98). IR (KBr) ν_max_ (cm^−1^): 3411, 2934, 1608, 1531, 1127 and 1009. ^1^H- and ^13^C-NMR data: Table 3 and Table 4. Negative ion HR-ESI-MS *m*/*z* 493.1665 [M − H]^−^ (calcd for C_31_H_26_O_6,_ 493.1657).

### 3.5. Cytotoxic Bioassay

MTT assays were performed to evaluate cytotoxic activities of all the compounds as previously reported [2]. Paclitaxel was served as a positive control. Data are presented as mean for three independent experiments. Each concentration of compounds was tested in three parallel wells. IC_50_ values were calculated using Microsoft Excel software.

## 4. Conclusions

Phytochemical investigations have led to the isolation of five new biphenanthrenes (**1**–**5**), together with six known ones (**6**–**11**), four of which (**6**–**9**) were reported from the genus *Cremastra* for the first time. 

The tubers of *C. appendiculata* are a famous traditional Chinese medicine with a long history for treating cancers. In this study, eleven biphenanthrenes have been obtained from the tubers of *C. appendiculata* and proved to have moderate or weak cytotoxicities to several cancer cell lines, which could account for the traditional usage of *C. appendiculata* as anti-cancer agent. Therefore, these biphenanthrenes might serve as chemical markers for quality control of this herb.

## Figures and Tables

**Table 1:** ^1^H-NMR Data of compounds **1**–**3**.

Proton	1 ^a^	2 ^b^	3 ^b^
1			6.71 s
3	6.97 s	7.00 s	
5	9.36 d (9.0)	9.38 d (9.0)	8.08 d (9.0)
6	7.09 dd (9.0, 3.0)	7.10 dd (9.0, 3.0)	6.63 dd (9.0, 3.0)
8	7.04 d (3.0)	7.06 d (3.0)	6.69 d (3.0)
9	7.28 d (9.0)	7.31 d (9.0)	2.73–2.78 m ^c^
10	6.90 d (9.0)	6.91 d (9.0)	2.73–2.78 m ^c^
3′	6.92 s	7.00 s	6.96 s
5′	8.96 s	9.17 d (9.0)	9.50 d (9.0)
6′		7.20 d (9.0)	7.17 dd (9.0 3.0)
8′	7.02 s		7.25 d (3.0)
9′	7.20 d (9.0)	7.66 d (9.0)	7.53 d (9.0)
10′	6.71 d (9.0)	6.98 d (9.0)	7.36 d (9.0)
4-OCH_3_	4.10 s	4.12 s	3.14 s
4′-OCH_3_	4.10 s	4.14 s	4.15 s
7′-OCH_3_			3.92 s
8′-OCH_3_		3.76 s	

^a^ ^1^H-NMR data were measured at 600 MHZ in DMSO-*d*_6_ for **1**, δ in ppm, *J* in Hz; ^b^ ^1^H-NMR data were measured at 500 MHZ in DMSO-*d*_6_ for **2**, in CD_3_OD for **3**, δ in ppm, *J* in Hz; ^c^ overlapped; the number in brackets represented coupling constants.

**Table 2:** ^13^C-NMR Data of compounds **1**–**3**.

Carbon	1 ^a^	2 ^b^	3 ^b^
1	111.3	111.0	112.3
2	153.3	153.5	156.0
3	99.7	99.7 ^**c**^	117.7
4	157.9	157.9	158.6
5	128.8	128.8	129.6
6	116.6	116.6	114.4
7	154.1	154.1	156.8
8	110.9	110.9	115.4
9	126.7	126.8	31.8
10	125.0	124.7	31.4
4a	114.2	114.2	121.1
4b	123.6	123.6	126.3
8a	132.5	132.4	140.9
10a	133.4	133.3	141.9
1′	110.6	111.0	110.9
2′	153.1	153.3	154.3
3′	99.0	99.8 ^c^	100.5
4′	157.7	157.8	160.5
5′	112.7	123.5	130.5
6′	145.6	117.4	117.2
7′	144.2	145.5	158.1
8′	111.7	140.8	109.5
9′	126.5	120.0	128.6
10′	121.5	124.7	126.5
4a′	113.8	114.2	116.7
4b′	124.4	124.3	126.6
8a′	125.6	126.2	134.5
10a′	133.8	133.4	135.2
4-OCH_3_	55.5	55.5	60.0
4′-OCH_3_	55.5	55.5	56.2
7′-OCH_3_			55.8
8′-OCH_3_		60.3	

^a^ ^13^C-NMR data were measured at 150 MHZ in DMSO-*d*_6_ for **1**, δ in ppm; ^b^ ^13^C-NMR data were measured at 125 MHZ in DMSO-*d*_6_ for **2**, in CD_3_OD for **3**, δ in ppm, *J* in Hz; ^c^ the signals under the same superscript may be interchanged.

**Table 3:** ^1^H-NMR Data of compounds **4**–**7**.

Proton	4	5	6	7
3	6.94 s	7.04 s	6.94 s	6.94 s
5	9.45 d (9.5)	9.51 d (9.5)	9.41 d (9.5)	9.41 d (9.5)
6	7.20 dd (9.5, 3.0)	7.24 dd (9.5, 3.0)	7.11 dd (9.5, 3.0)	7.11 dd (9.5, 3.0)
8	7.26 d (3.0)	7.37 d (3.0)	7.14 d (3.0)	7.13 d (3.0)
9	7.59 d (9.5)	7.70 d (9.5)	7.52 d (9.5)	7.50 d (9.5)
10	7.70 d (9.5)	7.74 d (9.5)	7.67 d (9.5)	7.66 d (9.5)
1′	6.67 d (3.0)	6.20 d (3.0)	6.24 d (3.0)	6.66 d (3.0)
3′	6.69 dd (9.5, 3.0)	6.65 d (3.0)	6.58 d (3.0)	6.67 dd (9.5, 3.0)
4′	8.05 d (9.5)			8.02 d (9.5)
5′		8.07 d (9.0)	8.02 d (9.5)	
6′	6.38 d (3.0)	6.69 dd (9.0,3.0)	6.61 dd (9.5, 3.0)	6.39 d (3.0)
8′	6.29 d (3.0)	6.68 d (3.0)	6.60 d (3.0)	6.29 d (3.0)
9′	2.61–2.63 m ^a^	2.59–2.60 m	2.59–2.63 m	2.61–2.63 m ^a^
10′	2.61–2.63 m ^a^	2.53–2.56 m	2.54–2.58 m	2.61–2.63 m ^a^
4-OCH_3_	4.12 s	4.16 s	4.12 s	4.12 s
7-OCH_3_	3.91 s	3.94 s		
4′-OCH_3_		3.81 s	3.75 s	
5′-OCH_3_	3.80 s			3.80 s

^1^H-NMR data were measured at 500 MHZ in CD_3_OD for **4**, **6**–**7**, in acetone-*d*_6_ for **5**, δ in ppm, *J* in Hz; ^a^ overlapped; the number in brackets represented coupling constants.

**Table 4:** ^13^C-NMR Data of compounds **4**–**7**.

Carbon	4	5	6	7
1	130.9	130.0	130.8	131.0
2	148.6	148.0	148.0	148.0
3	101.3	101.1	101.1	101.2
4	157.6	157.4	157.7	157.7
5	130.3	130.2	130.6	130.6
6	117.5	117.7	117.9	117.8
7	158.3	158.1	155.7	155.9
8	109.6	109.6	112.6	112.5
9	129.1	129.3	129.2	129.1
10	121.5	121.3	121.4	121.5
4a	116.4	116.1	116.8	116.8
4b	126.2	125.8	125.5	125.5
8a	134.4	134.1	134.4	134.8
10a	129.4	129.0	129.3	129.3
1′	114.7	107.3	107.9	114.9
2′	158.3	159.1	159.6	158.5
3′	113.5	99.6	99.6	113.6
4′	129.9	158.8	159.3	130.1
5′	159.3	130.2	130.4	159.3
6′	99.3	113.6	113.8	99.4
7′	158.0	156.5	156.6	158.0
8′	108.4	115.1	115.2	108.5
9′	31.6 ^a^	31.4 ^b^	30.8 ^c^	30.8 ^d^
10′	31.2 ^a^	30.6 ^b^	31.2 ^c^	31.4 ^d^
4a′	128.2	118.5	118.9	128.3
4b′	116.3	125.3	125.9	116.6
8a′	142.0	140.3	140.9	142.2
10a′	140.5	141.3	142.0	140.7
4-OCH_3_	56.3	56.4	56.4	56.4
7-OCH_3_	55.8	55.7		
4′-OCH_3_		56.0	56.1	
5′-OCH_3_	56.0			56.0

^13^C-NMR data were measured at 125 MHZ in CD_3_OD for **4**, **6**–**7**, in acetone-*d*_6_ for **5**, δ in ppm; ^a–d^ the signals under the same superscript may be interchanged.

**Table 5:** IC_50_ values (μmol/L) of compounds **1**–**11** against tumor cell lines.

Compounds	Cell Lines		
	HCT-116	Hela	MDA-MB-231
**1**	92.24 ± 8.41	38.08 ± 5.16	>100
**2**	63.62 ± 3.40	44.15 ± 4.31	65.55 ± 4.07
**3**	26.53 ± 6.14	19.94 ± 2.07	15.80 ± 3.31
**4**	33.03 ± 4.50	24.71 ± 3.21	56.14 ± 4.33
**5**	>100	39.17 ± 6.59	>100
**6**	41.15 ± 6.31	>100	>100
**7**	86.49 ± 9.59	>100	>100
**8**	32.33 ± 4.77	64.81 ± 5.72	45.46 ± 2.91
**9**	15.01 ± 1.90	>100	11.09 ± 2.89
**10**	14.05 ± 2.25	17.43 ± 3.07	13.86 ± 3.33
**11**	14.39 ± 0.26	13.23 ± 1.95	12.13 ± 0.38
paclitaxel	1.05 ± 0.23	0.09 ± 0.01	0.016 ± 0.003

## Supplementary Materials

Supplementary materials can be accessed at: http://www.mdpi.com/1420-3049/21/8/1089/s1.

## References

[1] Kovács A., Vasas A., Hohmann J. (2008). Natural phenanthrenes and their biological activity. Phytochemistry.

[2] Xue Z., Li S., Wang S.J., Wang Y.H., Yang Y.C., Shi J.G., He L. (2006). Mono-, Bi-, and triphenanthrenes from the tubers of *Cremastra appendiculata*. J. Nat. Prod..

[3] Wang Y., Guan S.H., Meng Y.H., Zhang Y.B., Cheng C.R., Shi Y.Y., Feng R.H., Zeng F., Wu Z.Y., Zhang J.X. (2013). Phenanthrenes, 9,10-dihydrophenanthrenes, bibenzyls, with their derivatives, and malate or tartrate benzyl ester glucosides from tubers of *Cremastra appendiculata*. Phytochemistry.

[4] Yang M.H., Cai L., Tai Z.G., Zeng X.H., Ding Z.T. (2010). Four new phenanthrenes from *Monomeria barbata* Lindl. Fitoterapia.

[5] Xu J.J., Yu H., Qing C., Zhang Y.L., Liu Y., Chen Y.G. (2009). Two new biphenanthrenes with cytotoxic activity from *Bulbophyllum odoratissimum*. Fitoterapia.

[6] Yao S., Tang C.P., Li X.Q., Ye Y. (2008). Phochinenins A–F, dimeric 9,10-dihydrophenanthrene derivatives, from *Pholidota chinensis*. Helv. Chim. Acta.

[7] Apel C., Dumontet V., Lozach O., Meijer L., Guéritte F., Litaudon M. (2012). Phenanthrene derivatives from *Appendicula reflexa* as new CDK1/cyclin B inhibitors. Phytochem. Lett..

[8] Majumder P.L., Bandyopadhyay S., Pal S. (2008). Rigidanthrin, a new dimeric phenanthrene derivative of the orchid *Bulbophyllum rigidum*. J. Indian Chem. Soc..

[9] Majumder P.L., Mukhoti N., Chattopadhyay S. (2008). Agrostophyllanthrol and isoagrostophyllanthrol, two novel diastereomeric phenanthropyran derivatives from the orchid *Agrostophyllum khasiyanum*. J. Indian Chem. Soc..

[10] Gutierrez R.M.P., Gonzalez A.M.N., Baez E.G., Diaz S.L. (2010). Studies on the constituents of bulbs of the orchid *Prosthechea michuacana* and antioxidant activity. Chem. Nat. Compd..

[11] Qian C.D., Jiang F.S., Yu H.S., Shen Y., Fu Y.H., Cheng D.Q., Gan L.S., Ding Z.S. (2015). Antibacterial biphenanthrenes from the Fibrous Roots of *Bletilla Striata*. J. Nat. Prod..

[12] Masae Y., Li B., Tomoko K., Keiko I., Shuzo T., Yuriko Y., Kenichi T. (1992). Bisphenanthreneethers from *Bletilla striata*. Phytochemistry.

[13] Liu L., Ye J., Li P., Tu P.F. (2014). Chemical constituents of tubers of *Cremastra appendiculata*. Chin. J. Chin. Mater. Med..

[14] Editorial Committee of the Administration Bureau of Traditional Chinese Medicine (1999). Chinese Materia Medica (Zhong Hua Ben Cao).

[15] Liu L., Li J., Zeng K.W., Li P., Tu P.F. (2013). Three new phenanthrenes from *Cremastra appendiculata* (D. Don) Makino. Chin. Chem. Lett..

[16] Liu L., Li J., Zeng K.W., Li P., Tu P.F. (2015). Five new benzylphenanthrenes from *Cremastra appendiculata*. Fitoterapia.

