# Supplementary material for: Five New Biphenanthrenes from Cremastra appendiculata
Source: Molecules. 2016 Aug 19;21(8):1089. doi: 10.3390/molecules21081089 (PMC6272948; doi:10.3390/molecules21081089)
Supplement: Supplementary file 1 [file molecules-21-01089-s001.pdf]

Supplementary Materials: Five New Biphenanthrenes from *Cremastra appendiculata*

Liang Liu, Jun Li , Ke-Wu Zeng , Yong Jiang , Peng-Fei Tu

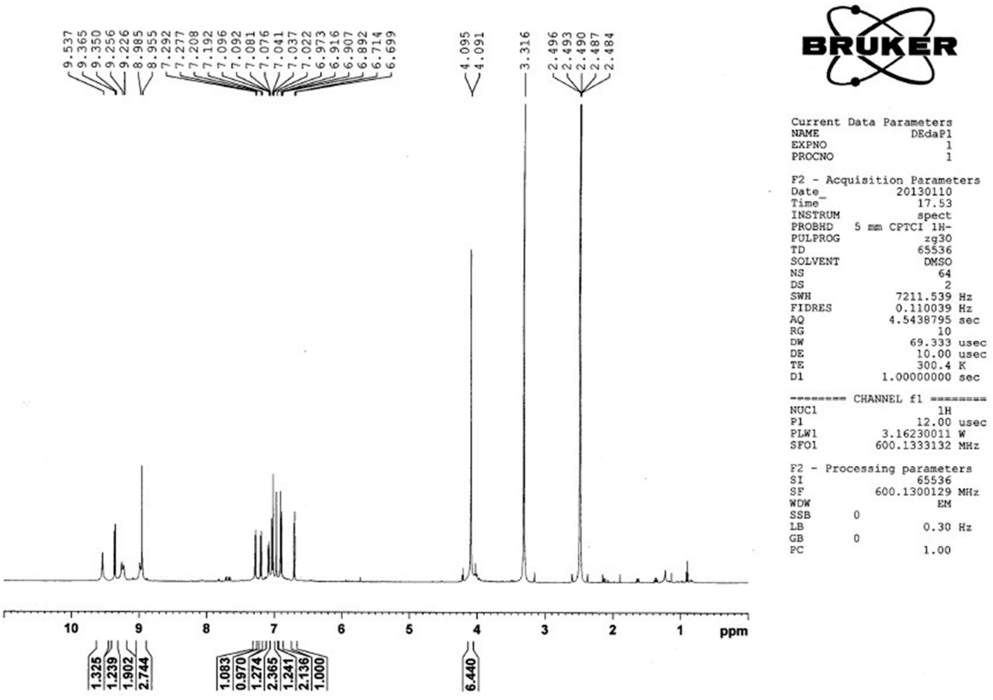

Figure S1. ^{1}H-NMR spectrum of compound **1**.

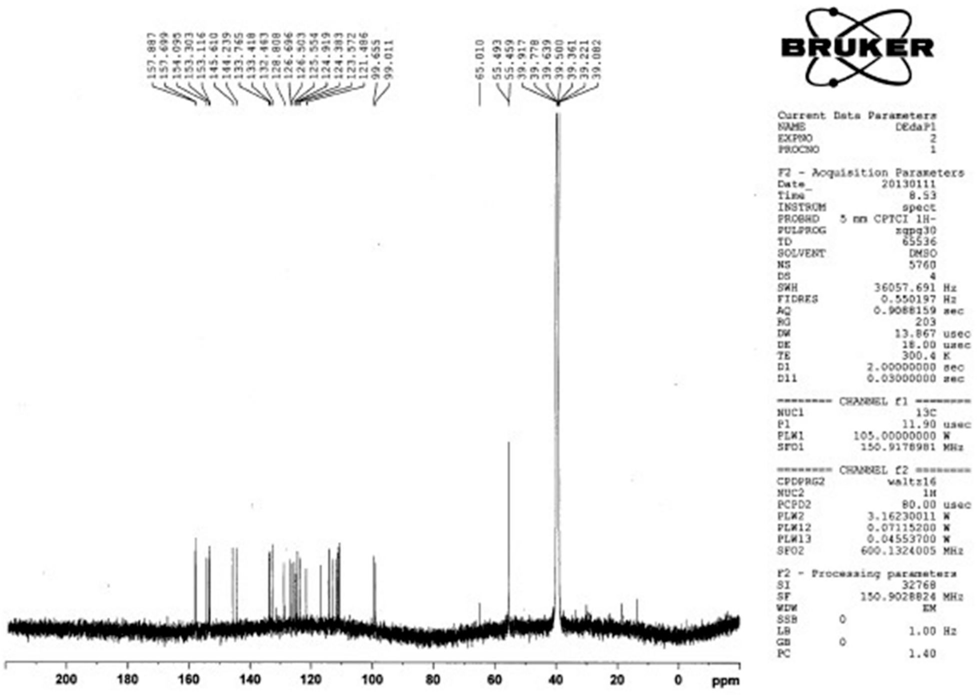

Figure S2. ^{13}C-NMR spectrum of compound **1**.

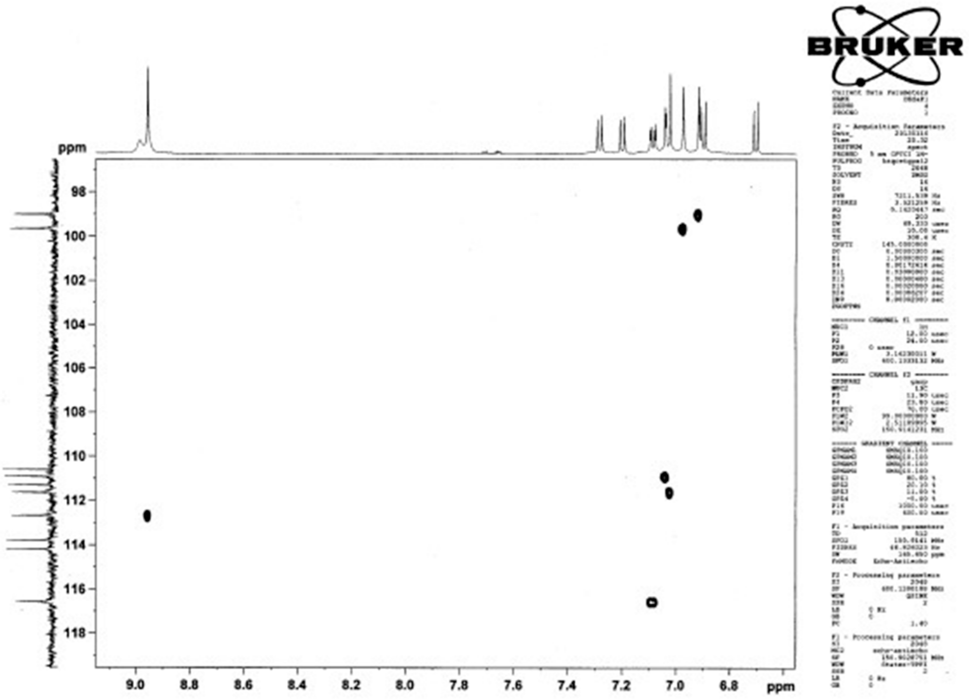

Figure S3. HSQC spectrum of compound **1**.

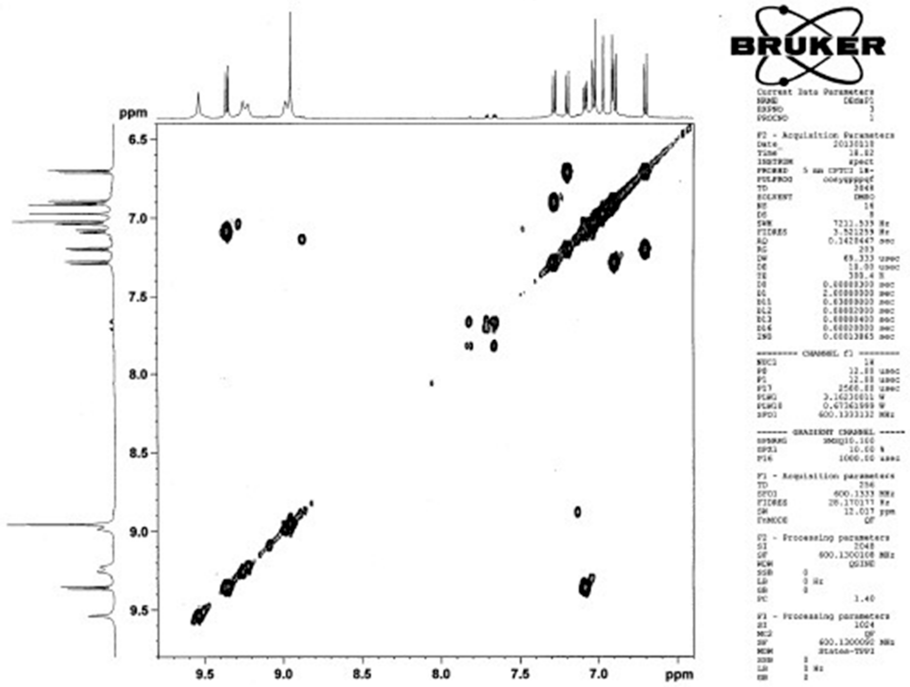

Figure S4. ^{1}H-^{1}H COSY spectrum of compound **1**.

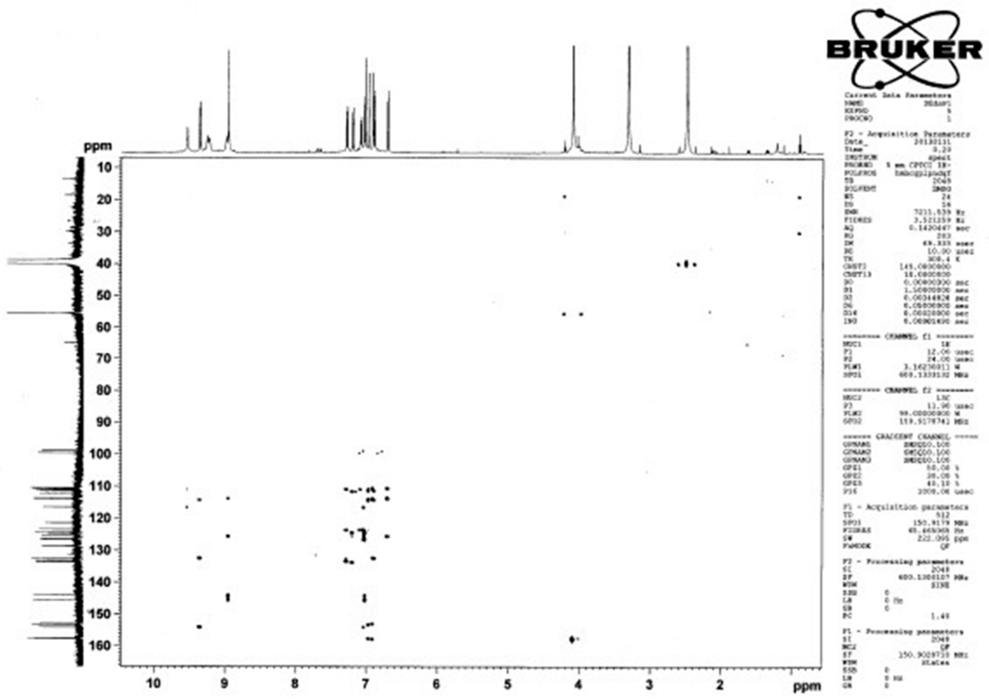

Figure S5. HMBC spectrum of compound **1**.

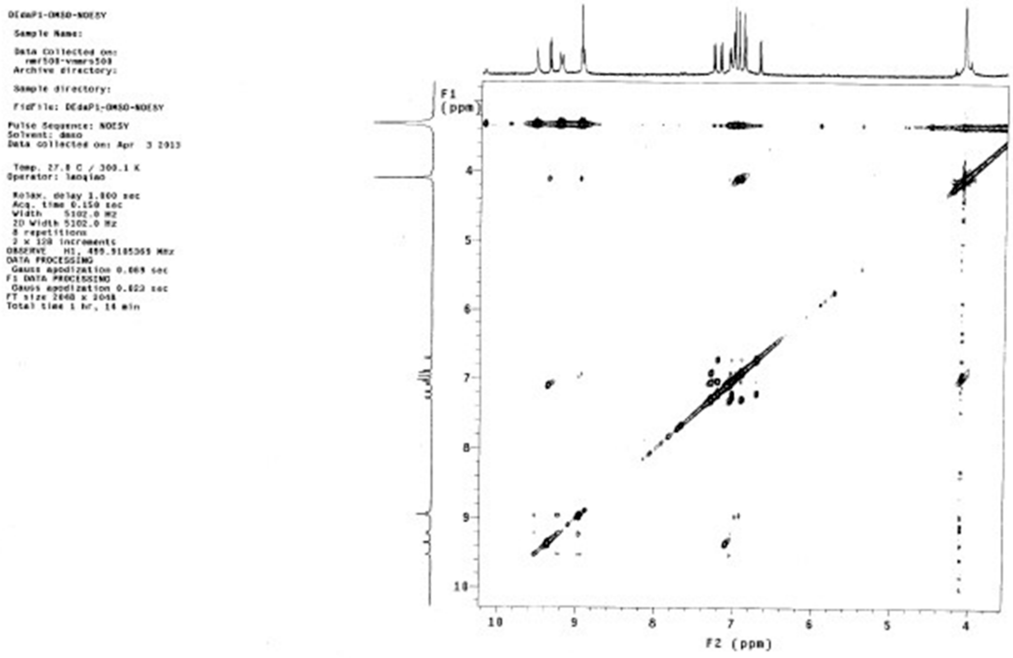

Figure S6. NOESY spectrum of compound **1**.

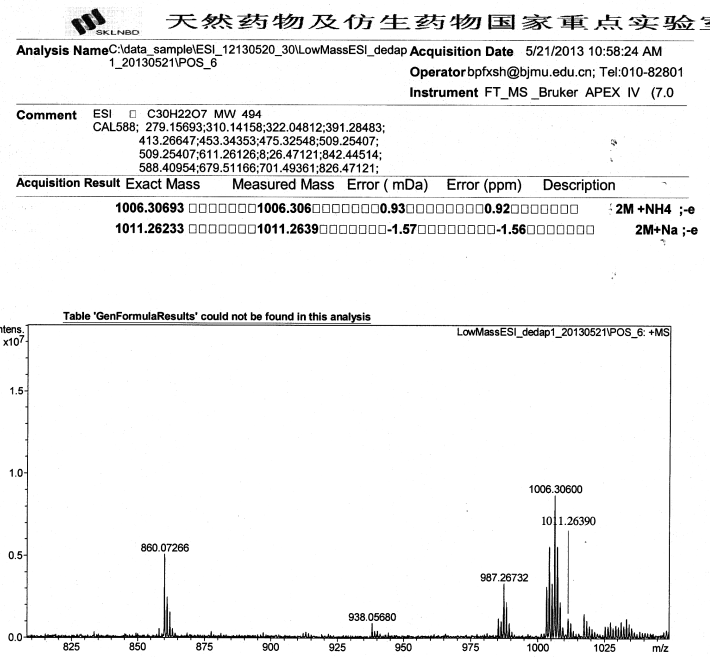

Figure S7. HR-ESI-MS spectrum of compound **1**.

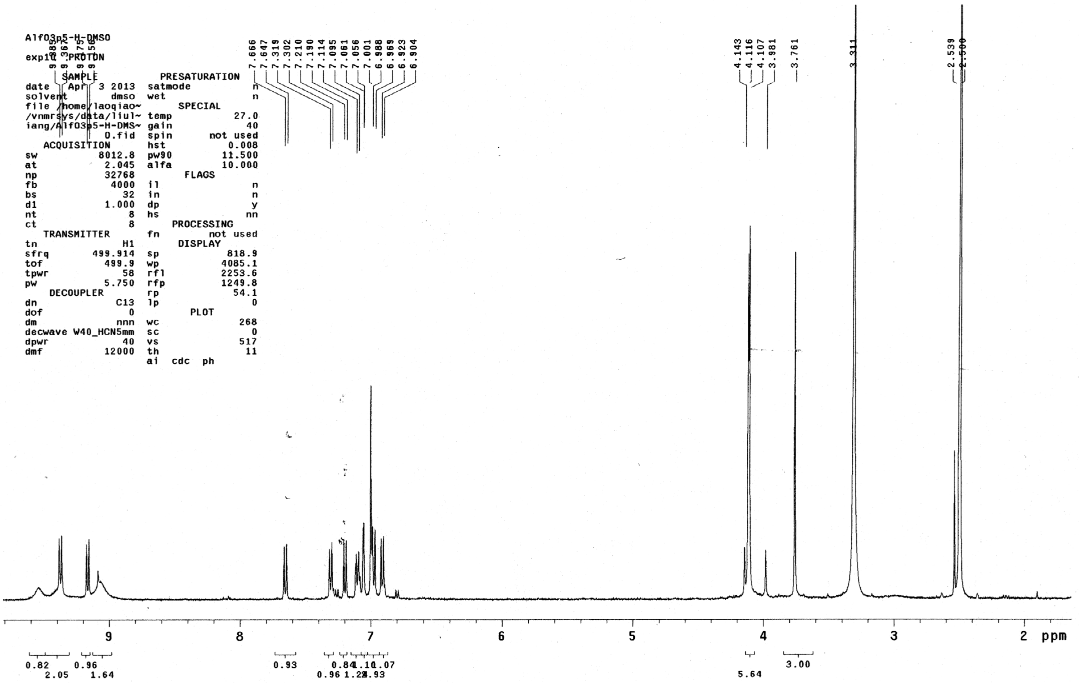

Figure S8. ^{1}H-NMR spectrum of compound **2**.

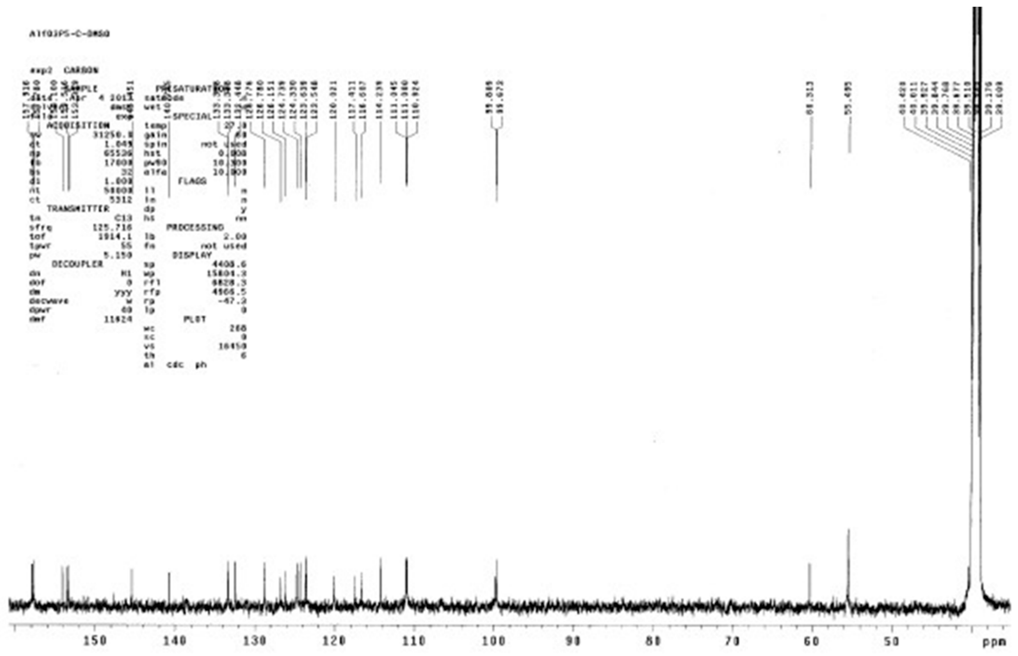

Figure S9. ^{13}C-NMR spectrum of compound **2**

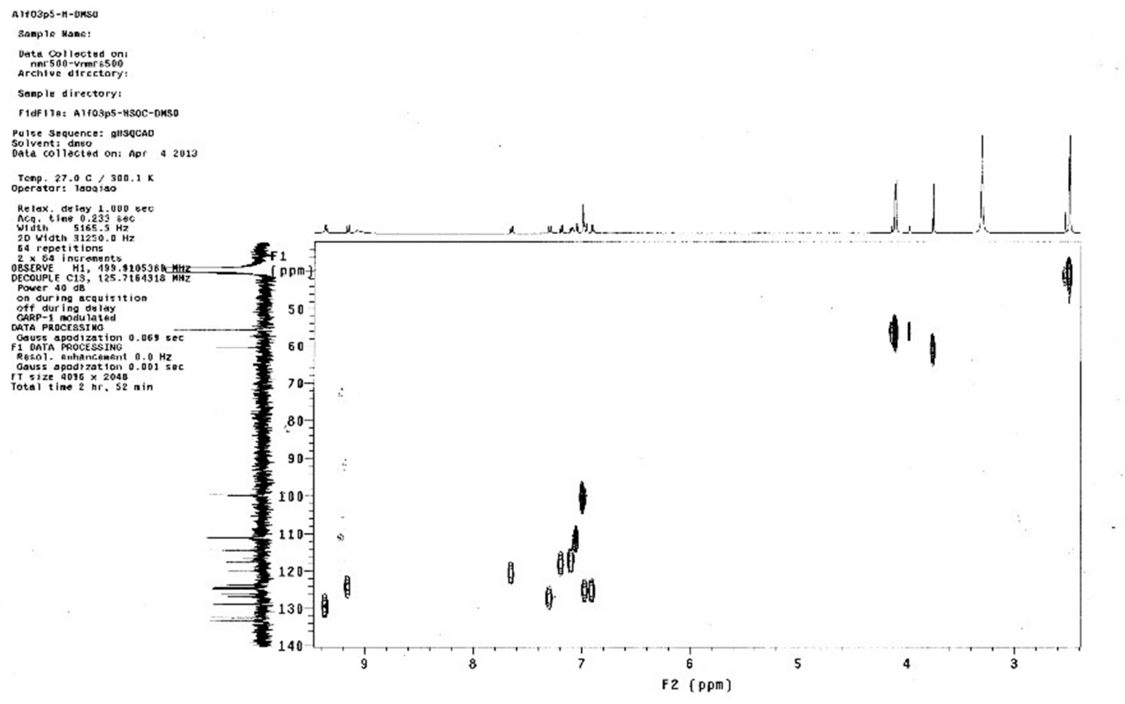

Figure S10. HSQC spectrum of compound **2**.

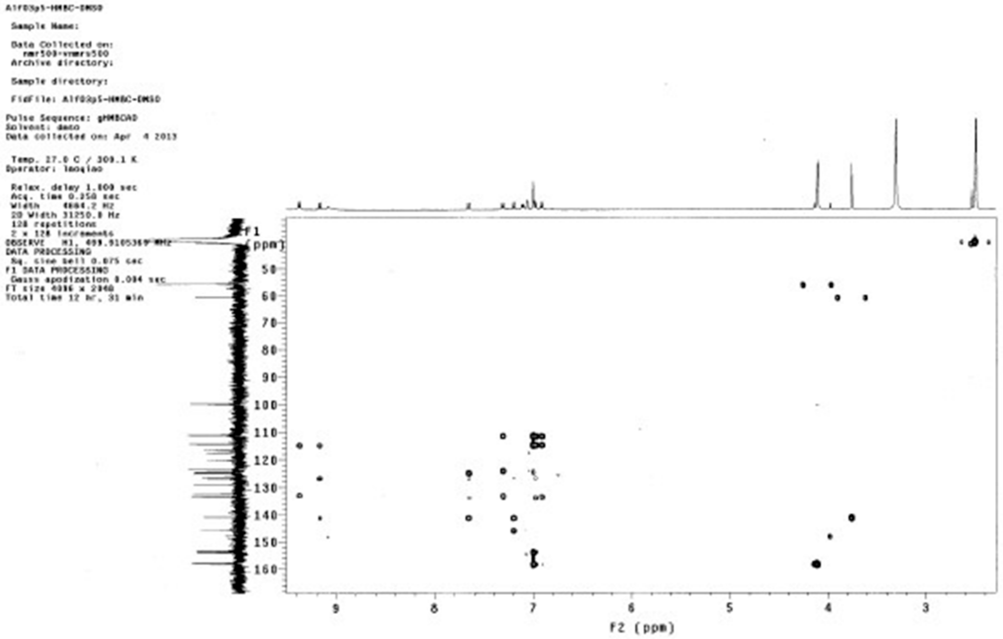

Figure S11. HMBC spectrum of compound **2**.

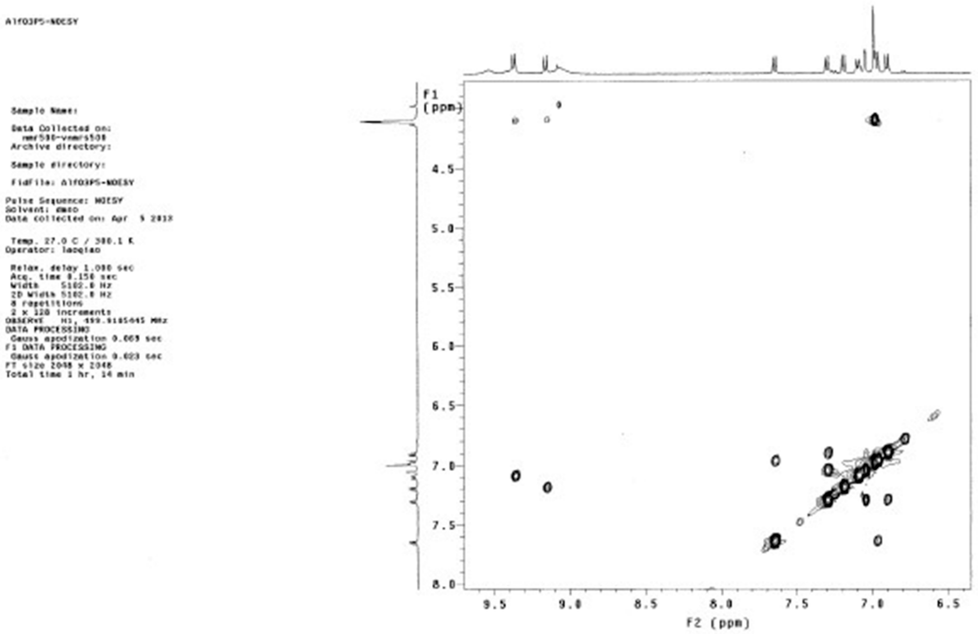

Figure S12. NOESY spectrum of compound **2**.

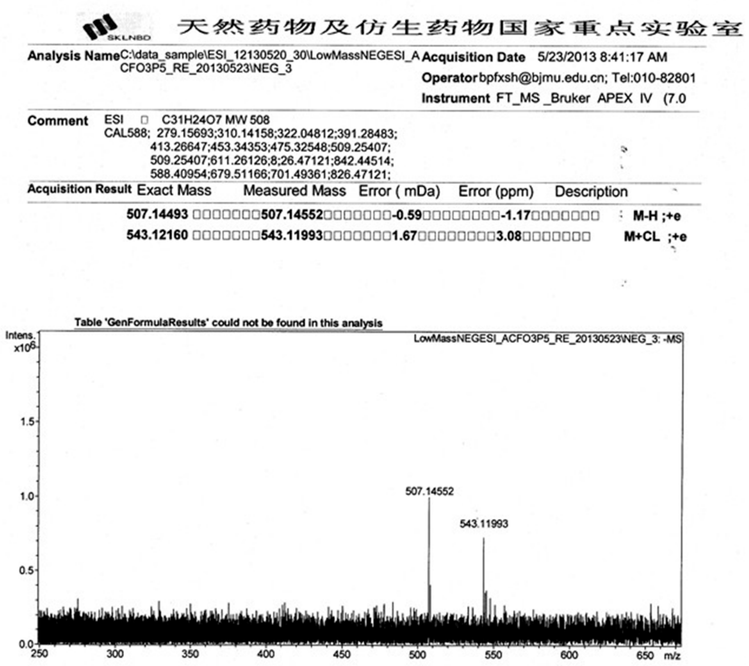

Figure S13. HR-ESI-MS spectrum of compound **2**.

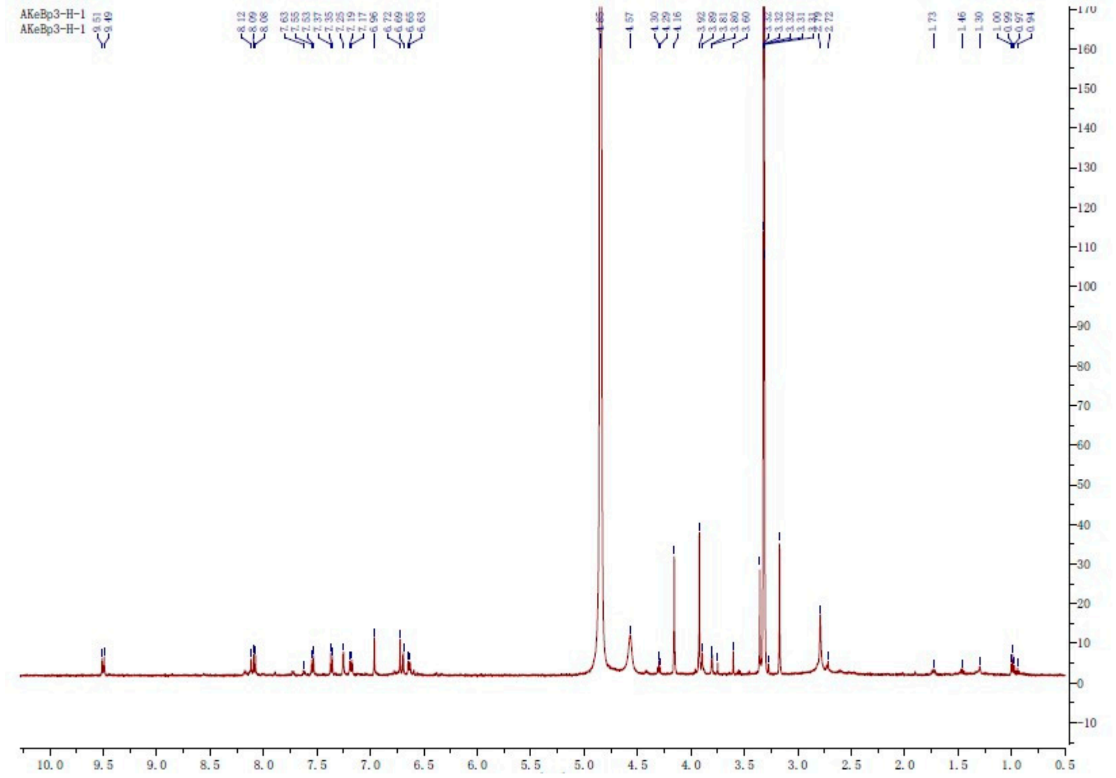

Figure S14. ^{1}H-NMR spectrum of compound **3**.

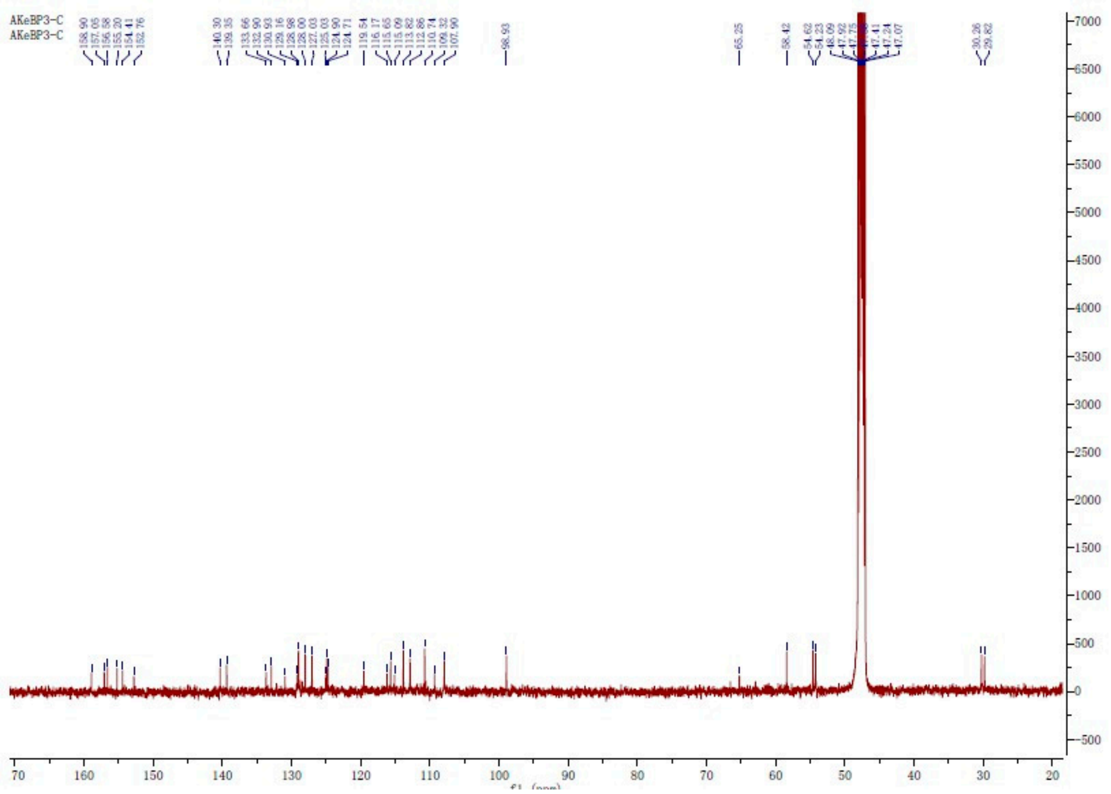

Figure S15. ^{13}C-NMR spectrum of compound **3**.

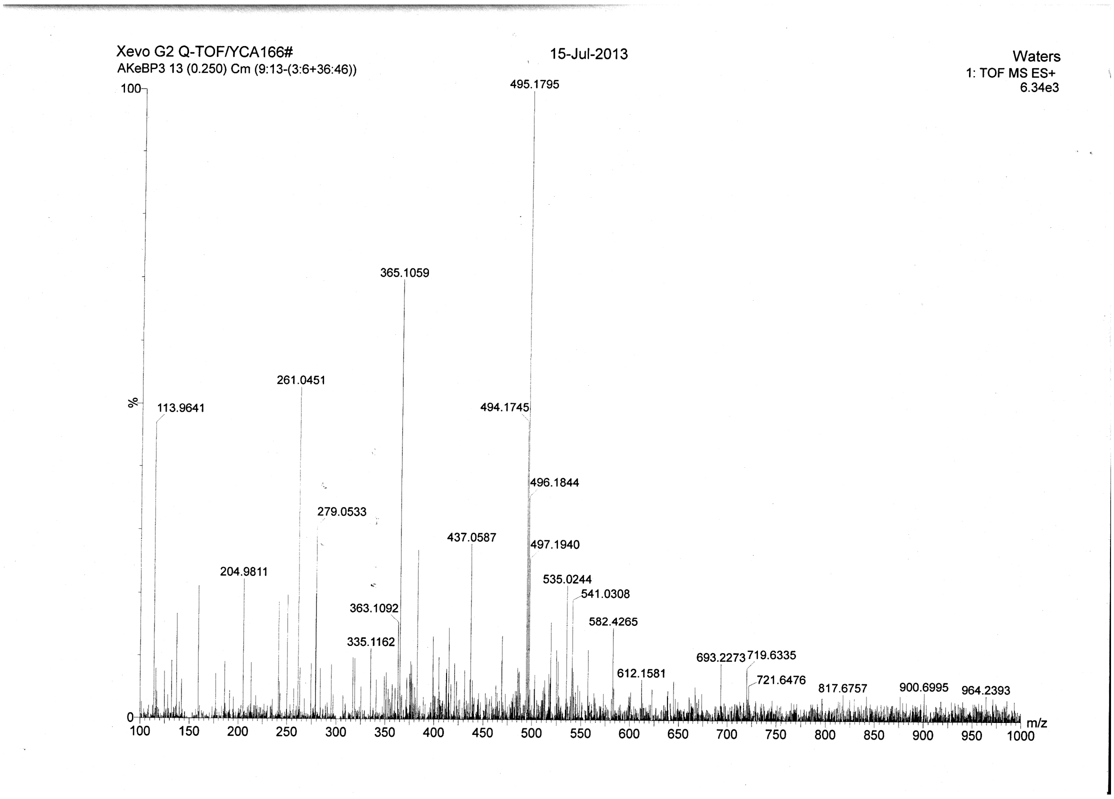

Figure S16. HR-ESI-MS spectrum of compound **3**.

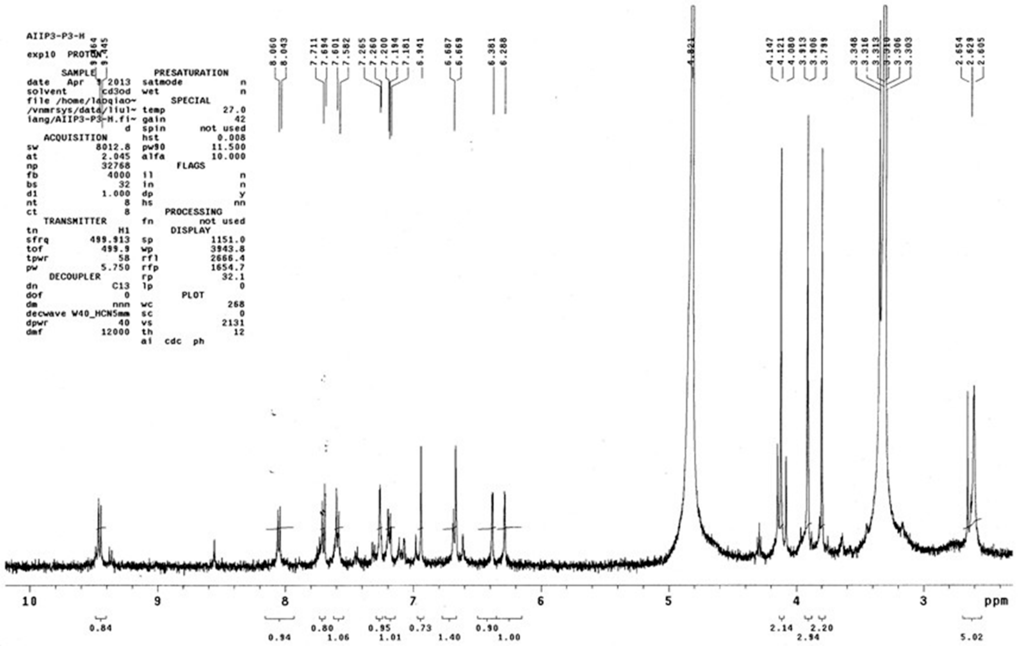

Figure S17. ^{1}H-NMR spectrum of compound **4**.

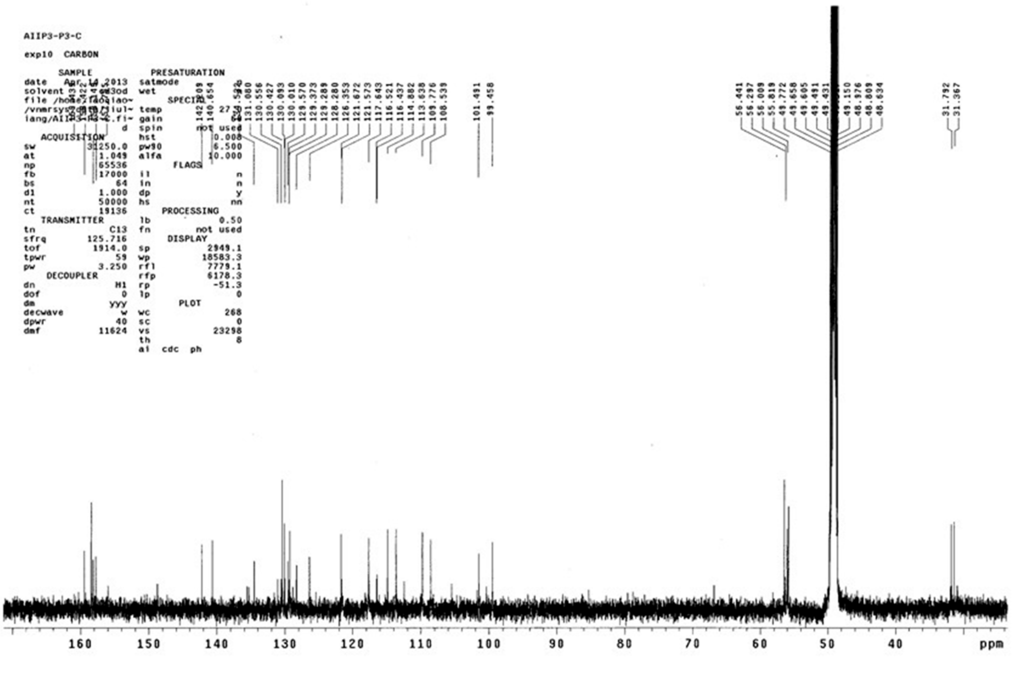

Figure S18. ^{13}C-NMR spectrum of compound **4**.

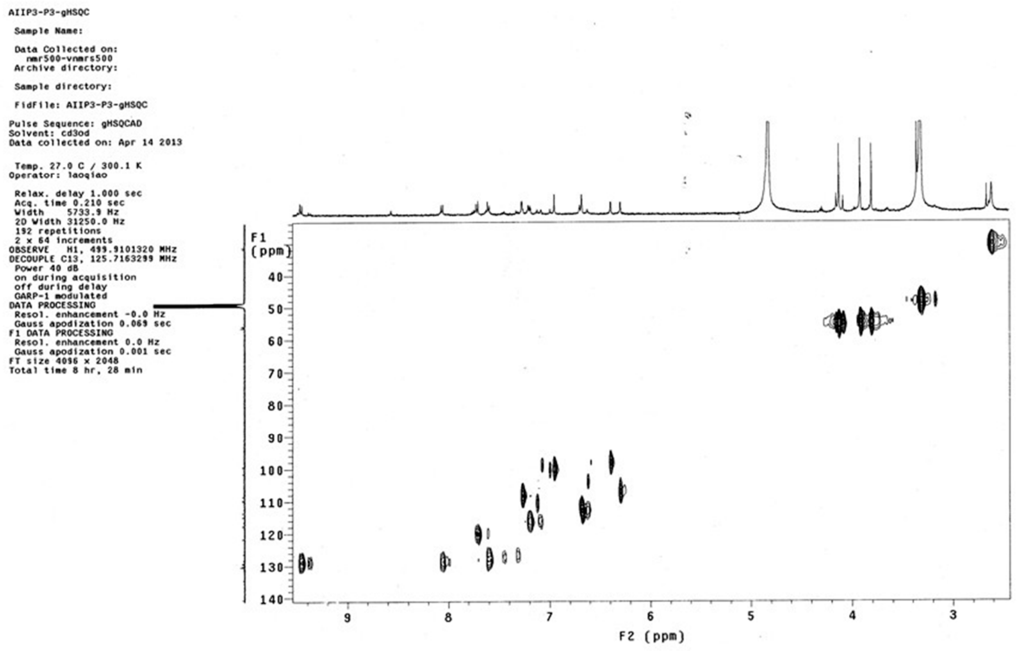

Figure S19. HSQC spectrum of compound **4**.

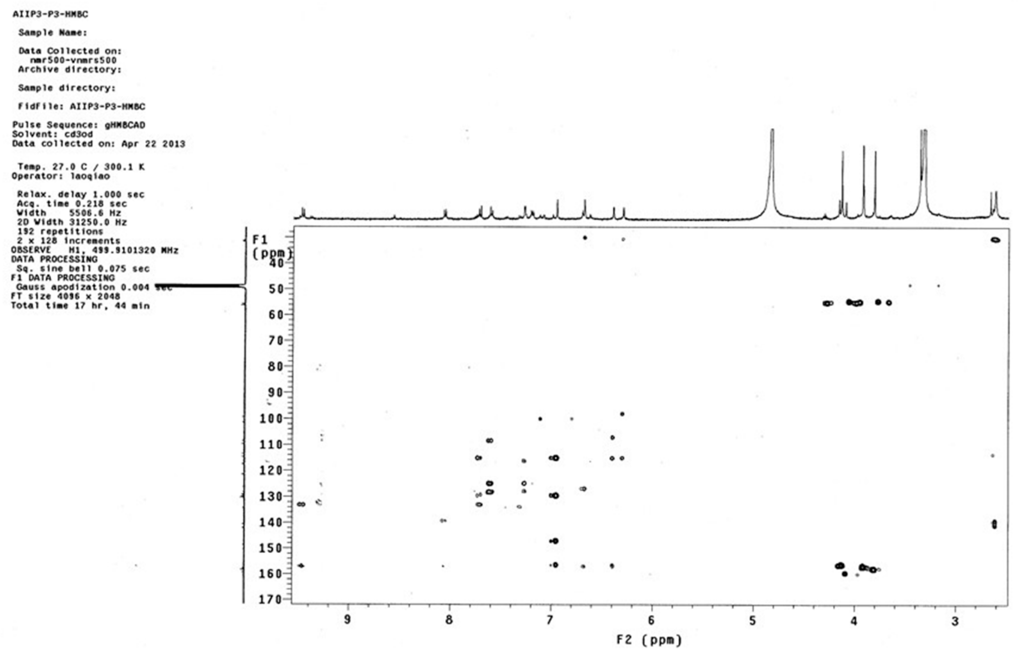

Figure S20. HMBC spectrum of compound **4**.

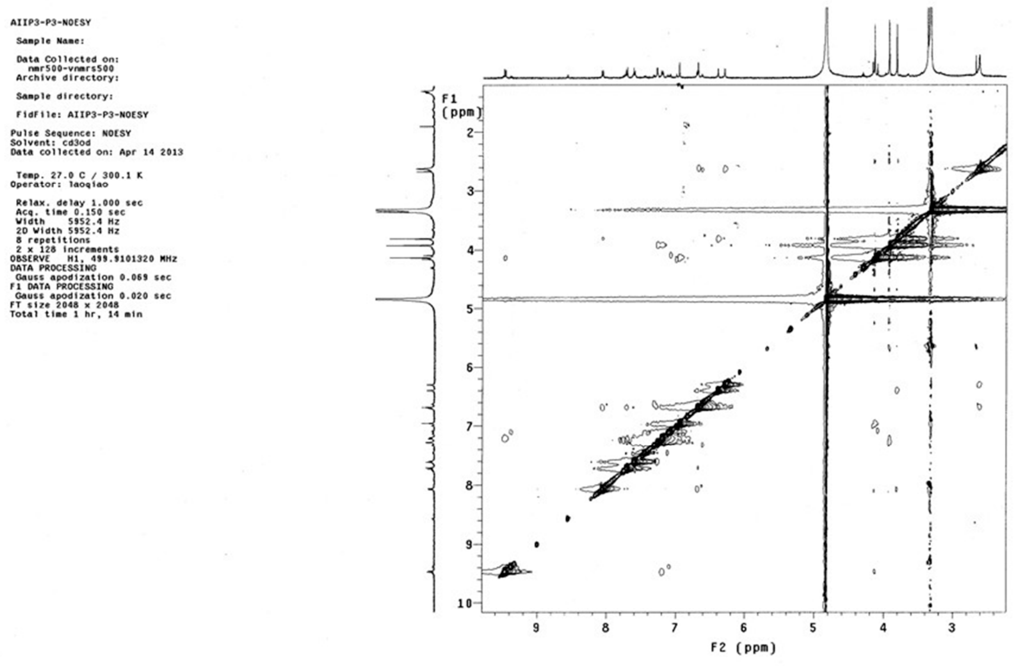

Figure S21. NOESY spectrum of compound **4**.

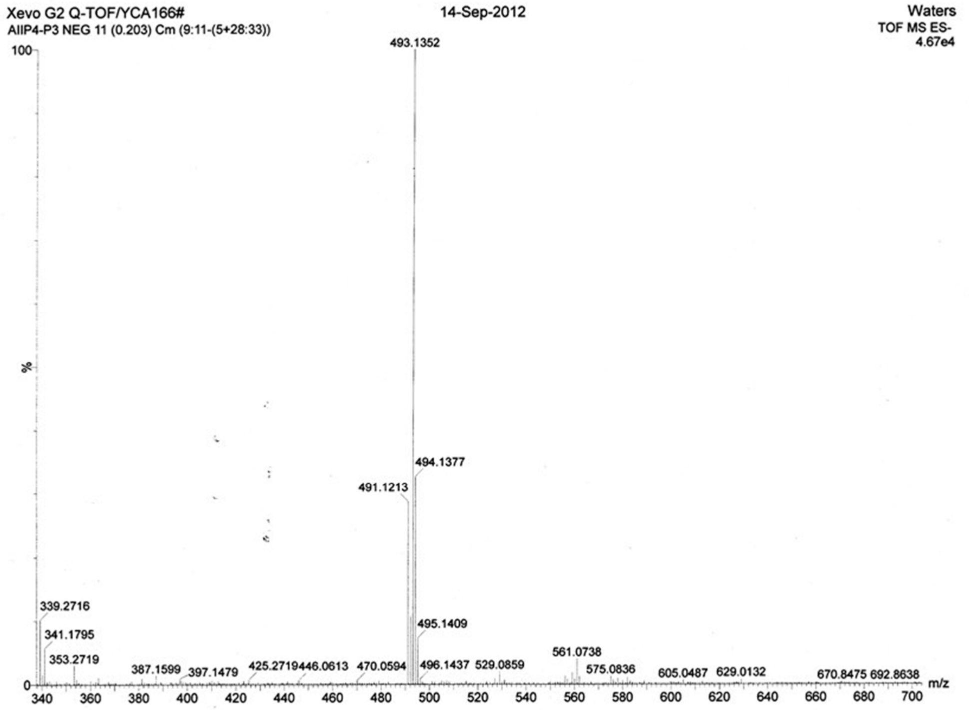

Figure S22. ESI-MS spectrum of compound **4**.

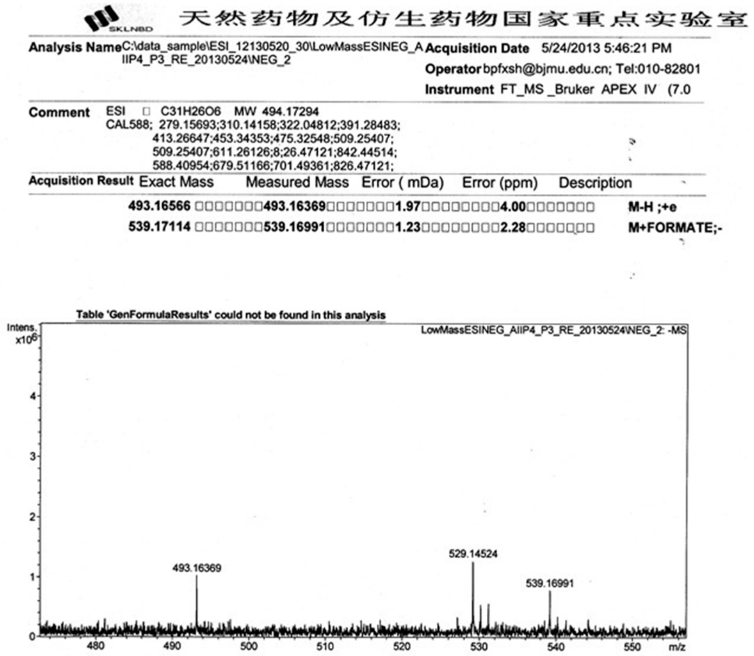

Figure S23. HR-ESI-MS spectrum of compound **4**.

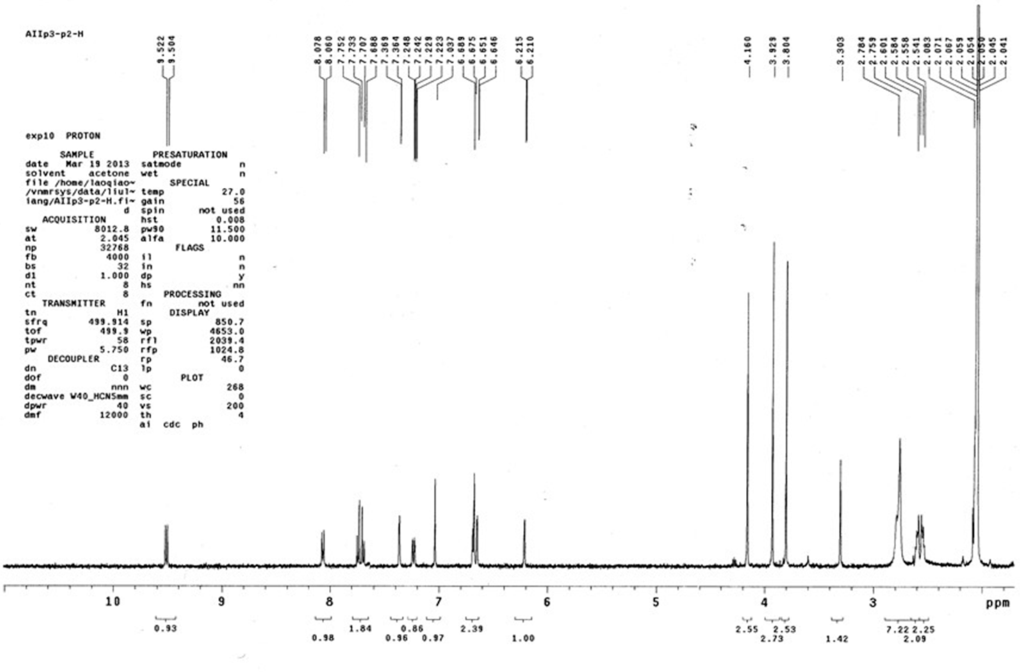

Figure S24. ^{1}H-NMR spectrum of compound **5**.

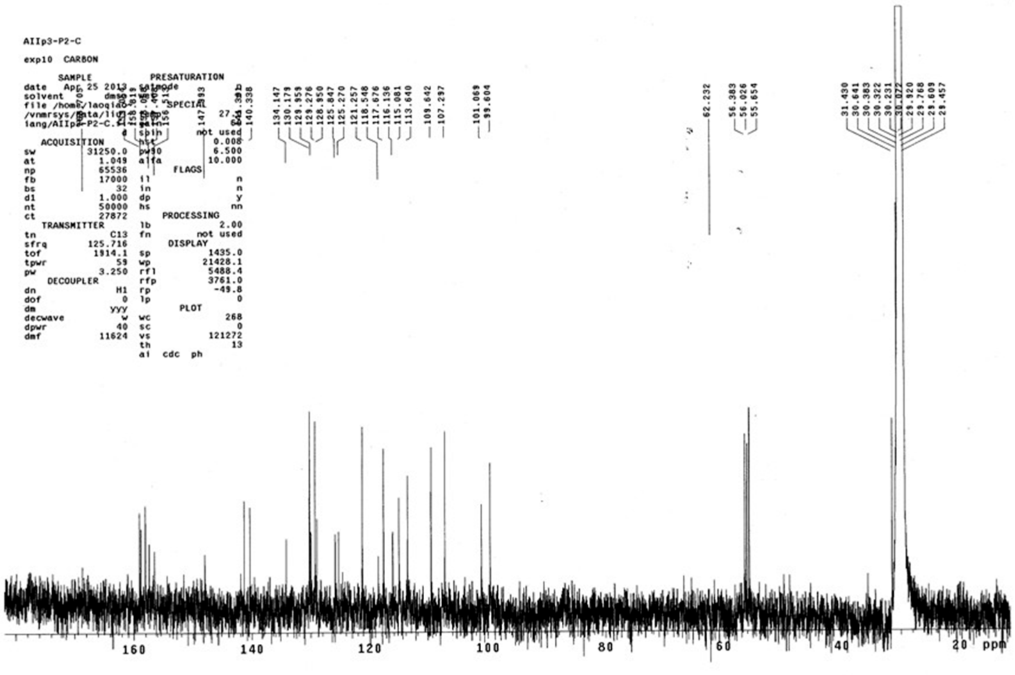

Figure S25. ^{13}C-NMR spectrum of compound **5**.

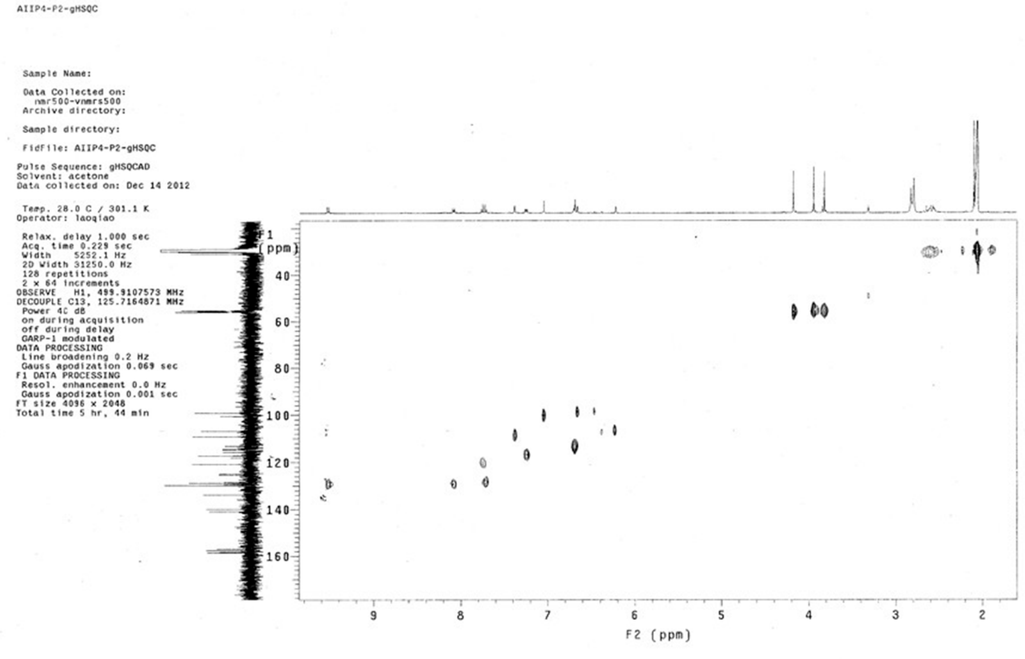

Figure S26. HSQC spectrum of compound **5**.

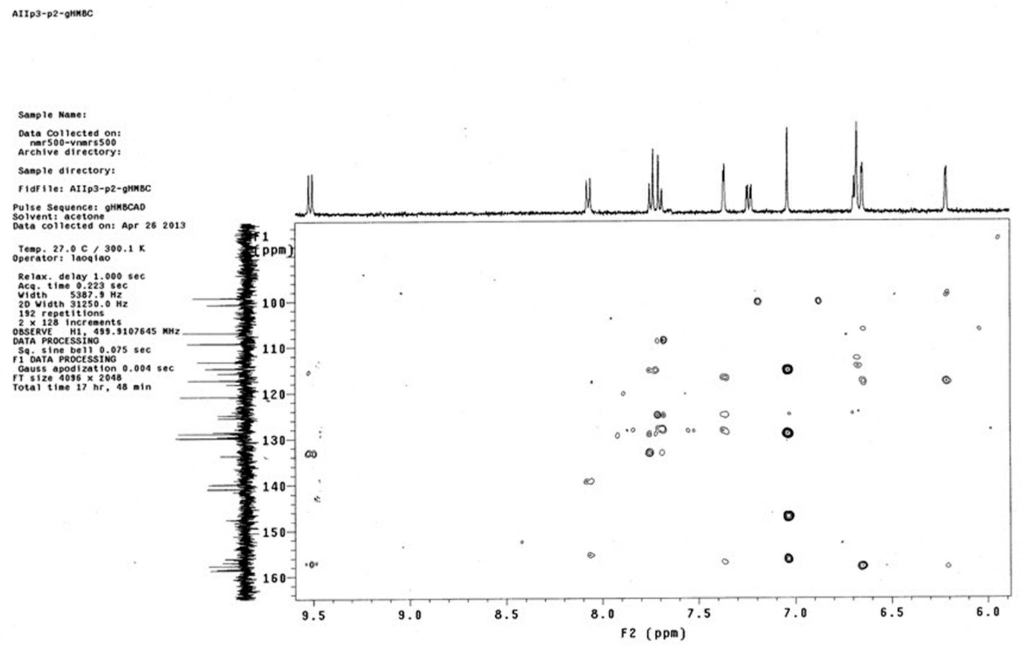

Figure S27. HMBC spectrum of compound **5**.

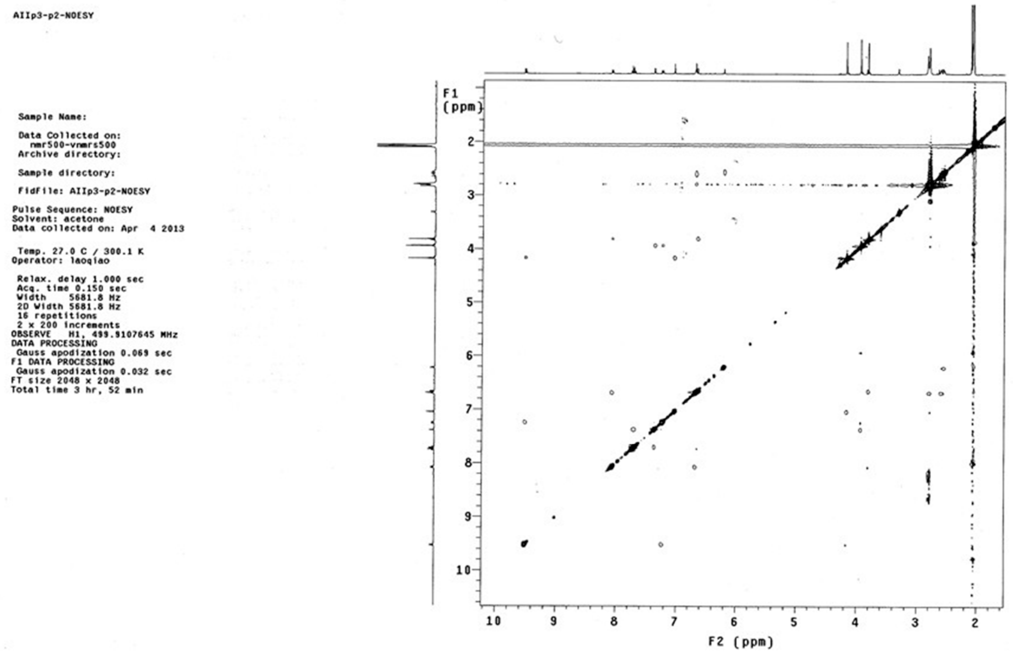

Figure S28. NOESY spectrum of compound **5**.

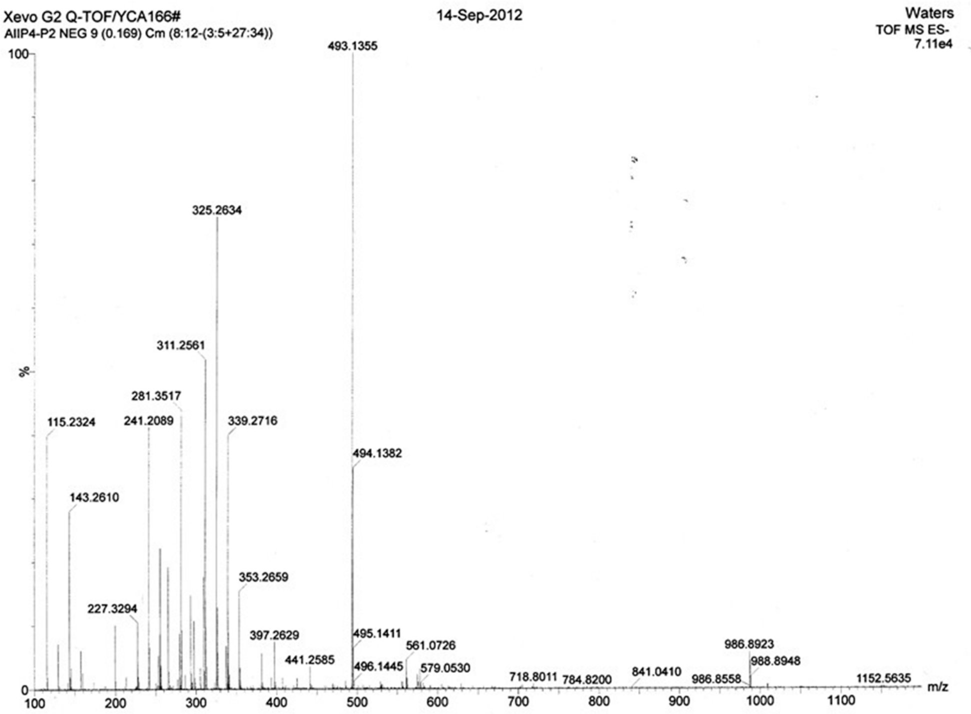

Figure S29. ESI-MS spectrum of compound **5**.

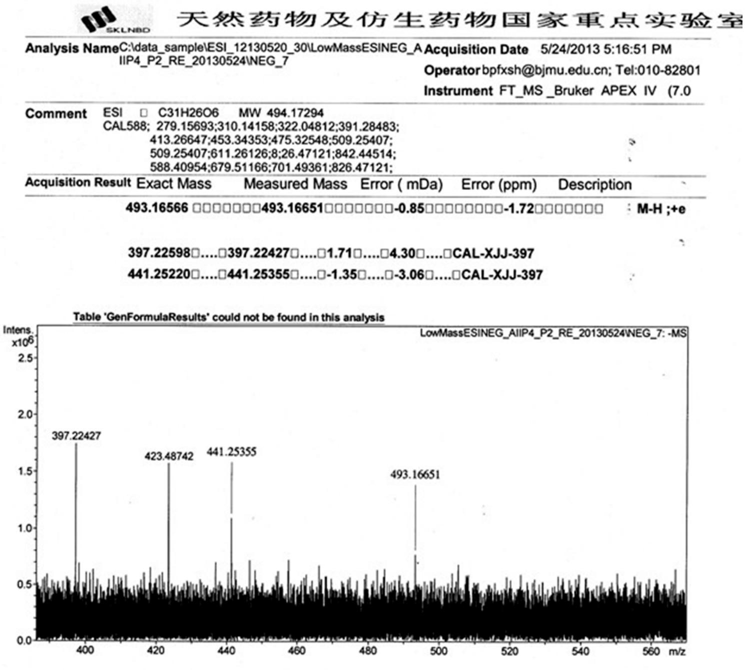

Figure S30. HR-ESI-MS spectrum of compound **5**.

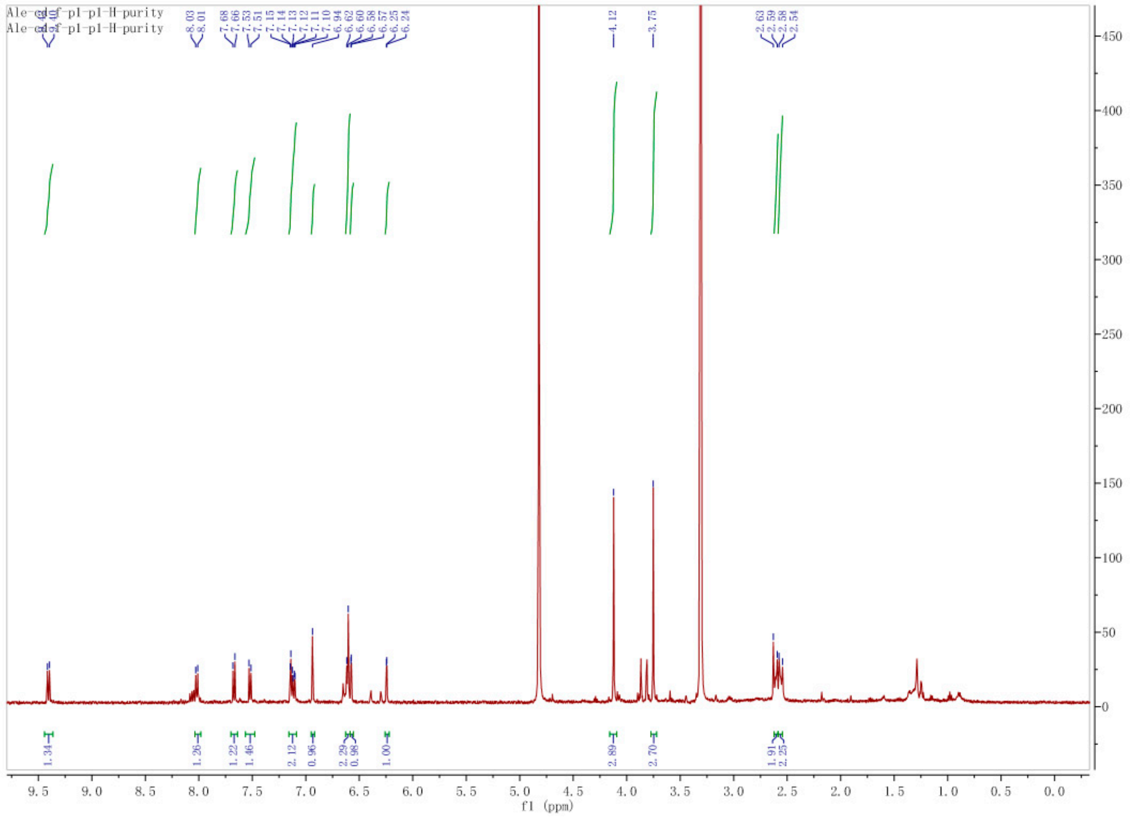

Figure S31. ^{1}H-NMR spectrum of compound **6**.

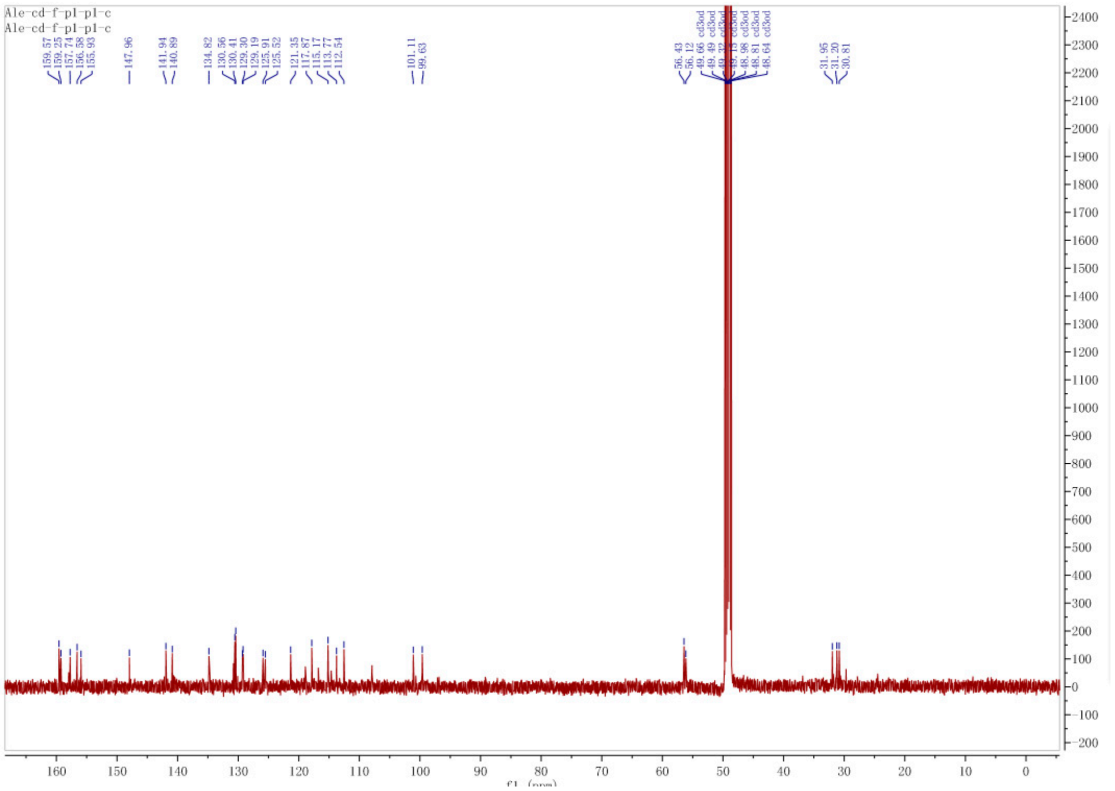

Figure S32. ^{13}C-NMR spectrum of compound **6**.

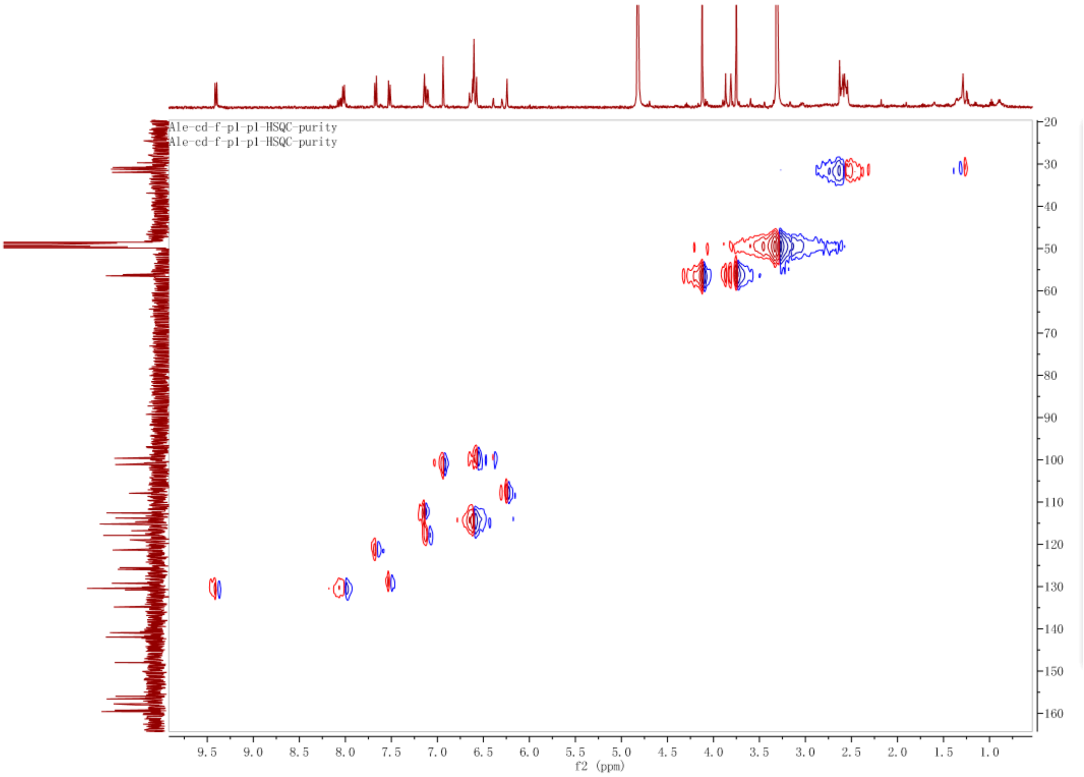

Figure S33. HSQC spectrum of compound **6**.

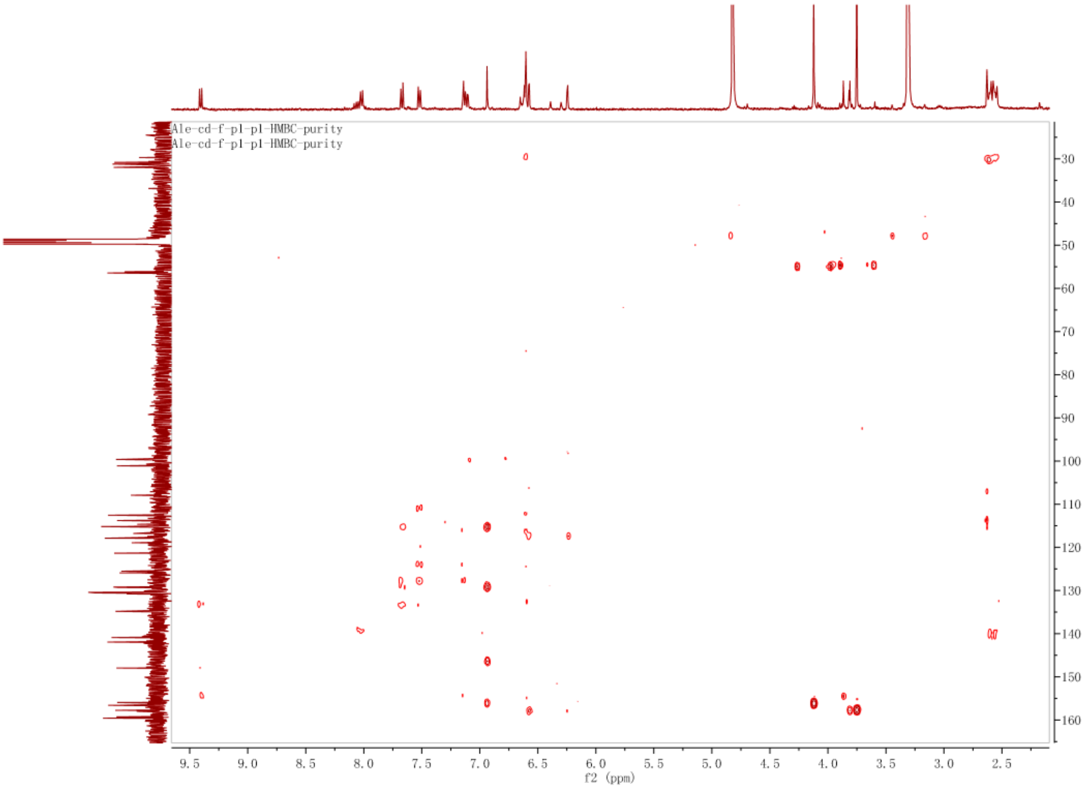

Figure S34. HMBC spectrum of compound **6**.

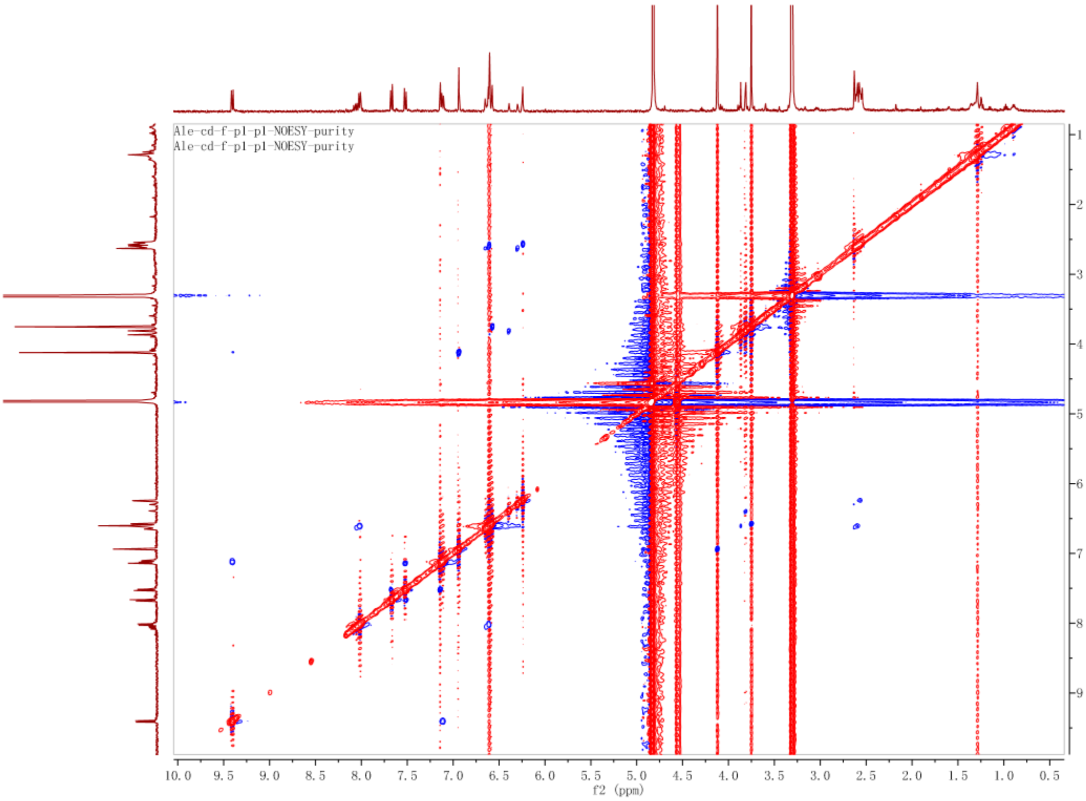

Figure S35. NOESY spectrum of compound **6**.

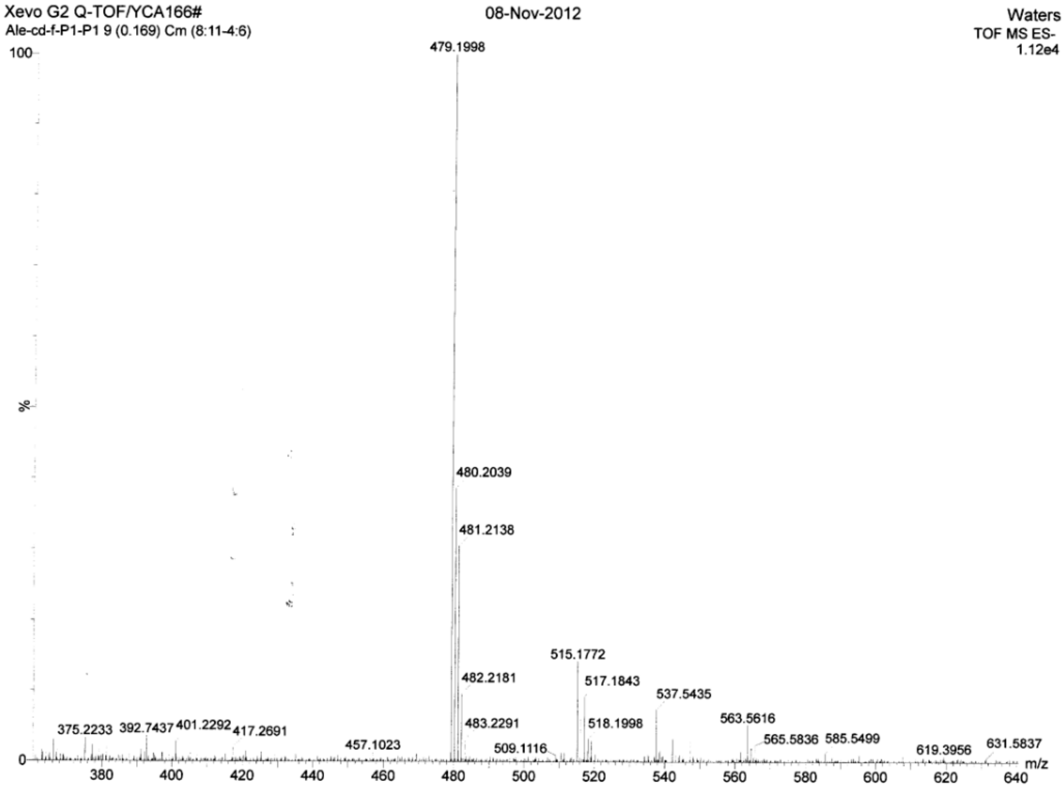

Figure S36. ESI-MS spectrum of compound **6**.

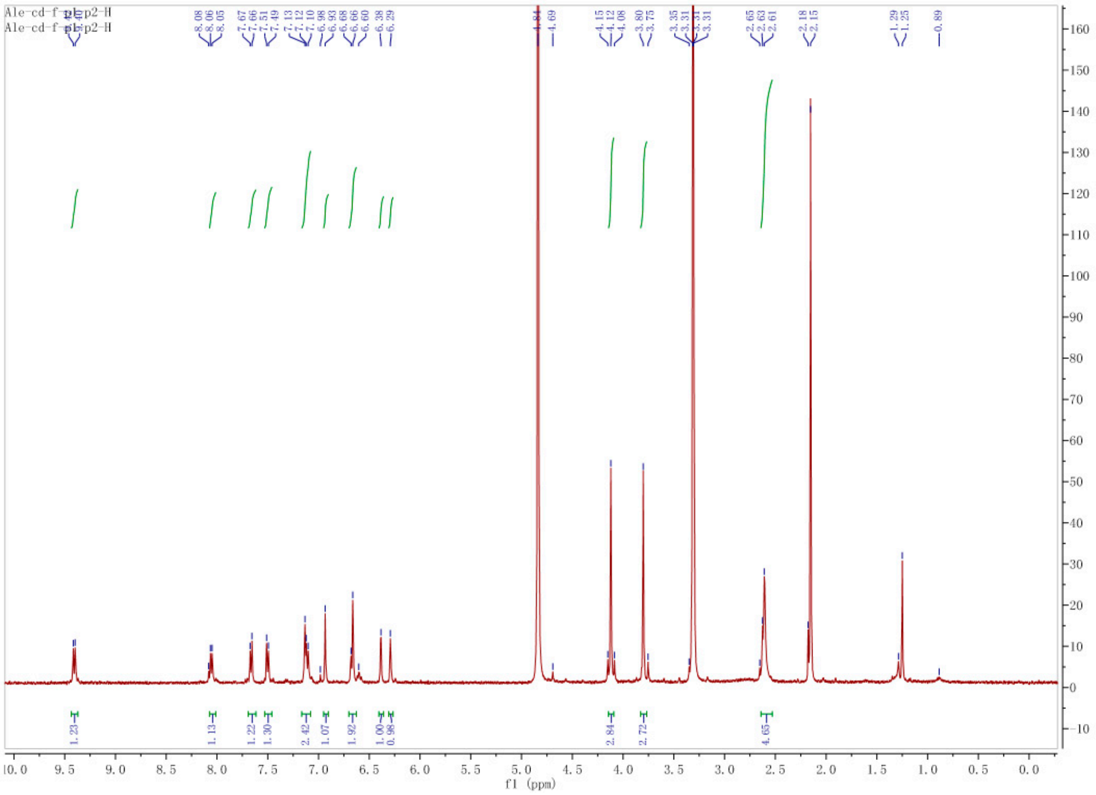

Figure S37. ^{1}H-NMR spectrum of compound **7**.

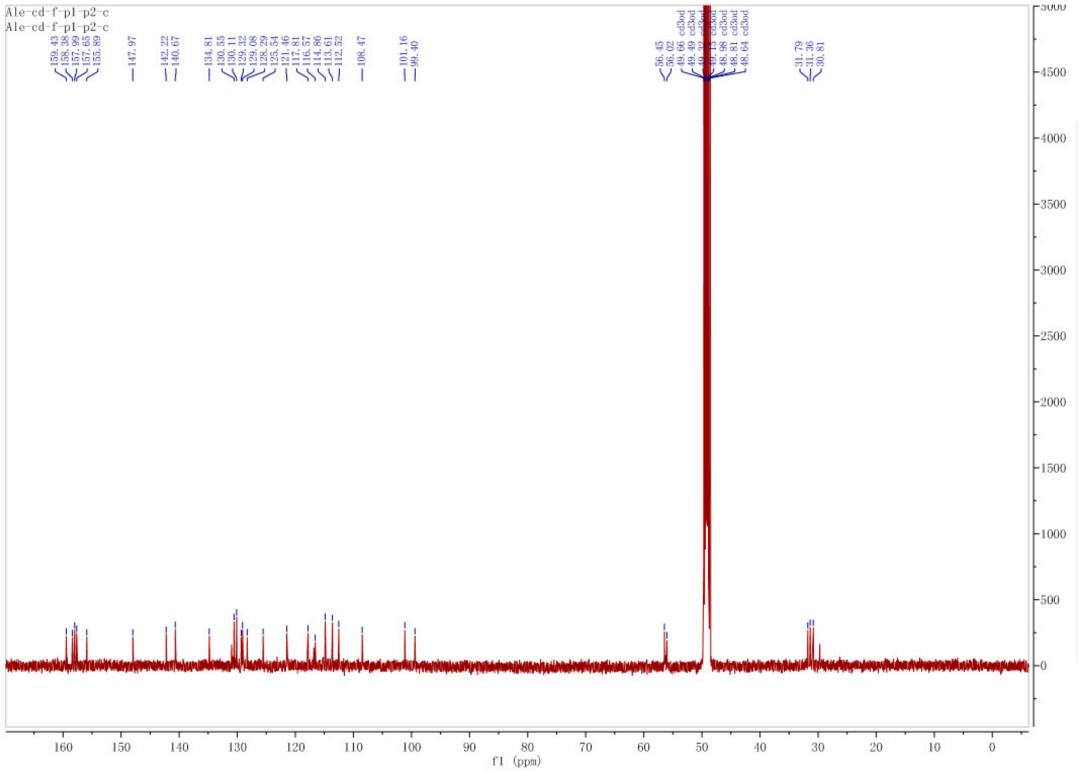

Figure S38. ^{13}C-NMR spectrum of compound **7**.

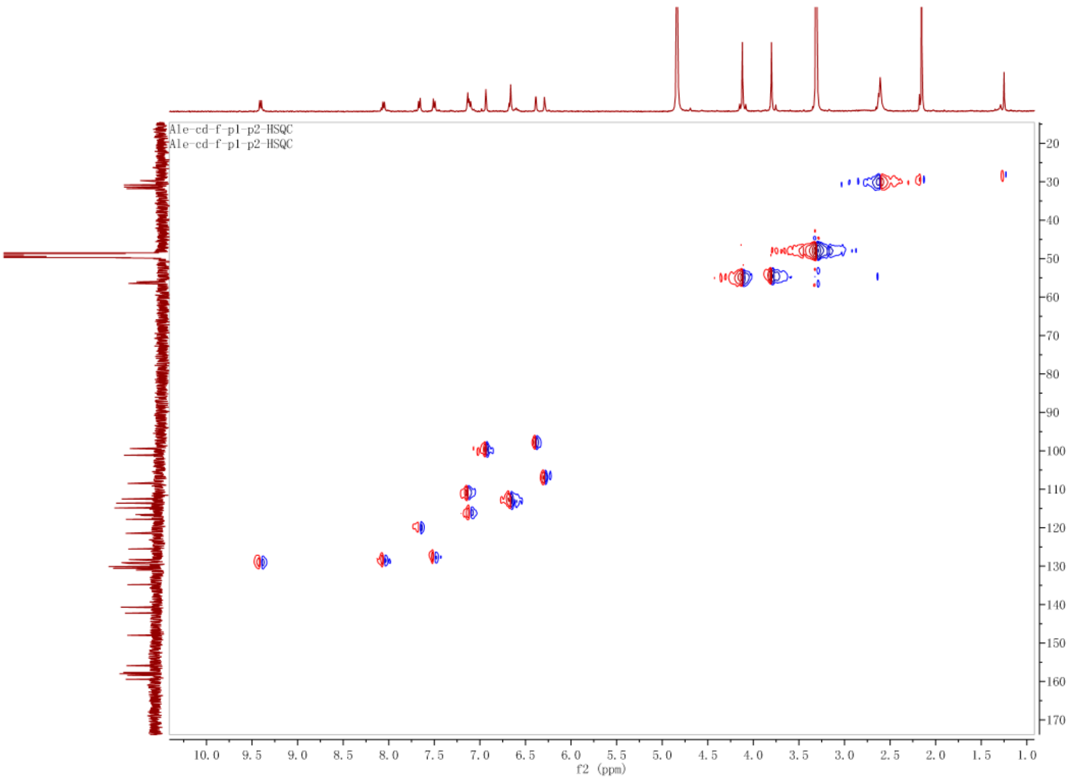

Figure S39. HSQC spectrum of compound **7**.

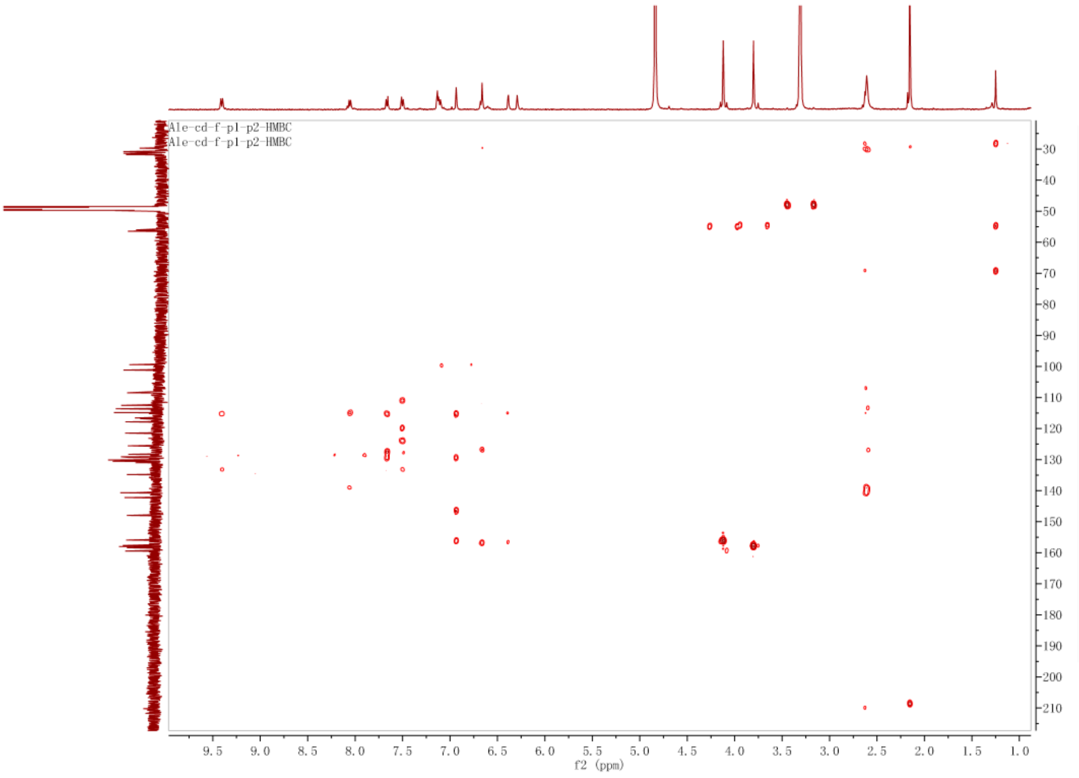

Figure S40. HMBC spectrum of compound **7**.

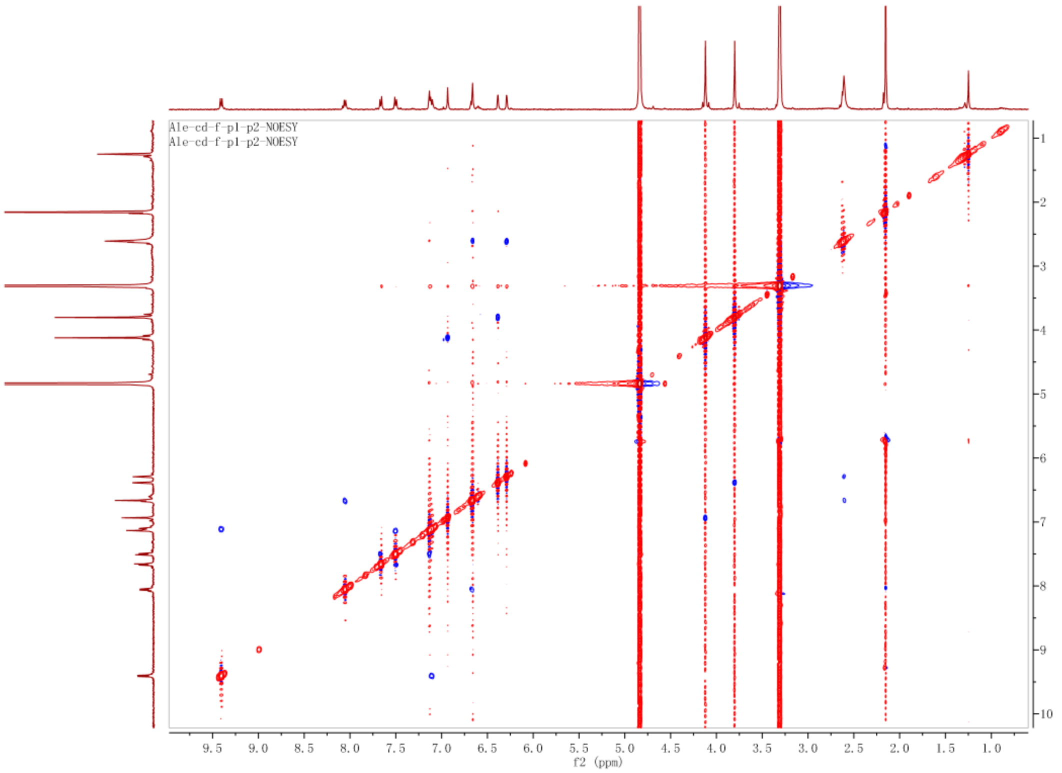

Figure S41. NOESY spectrum of compound **7**.

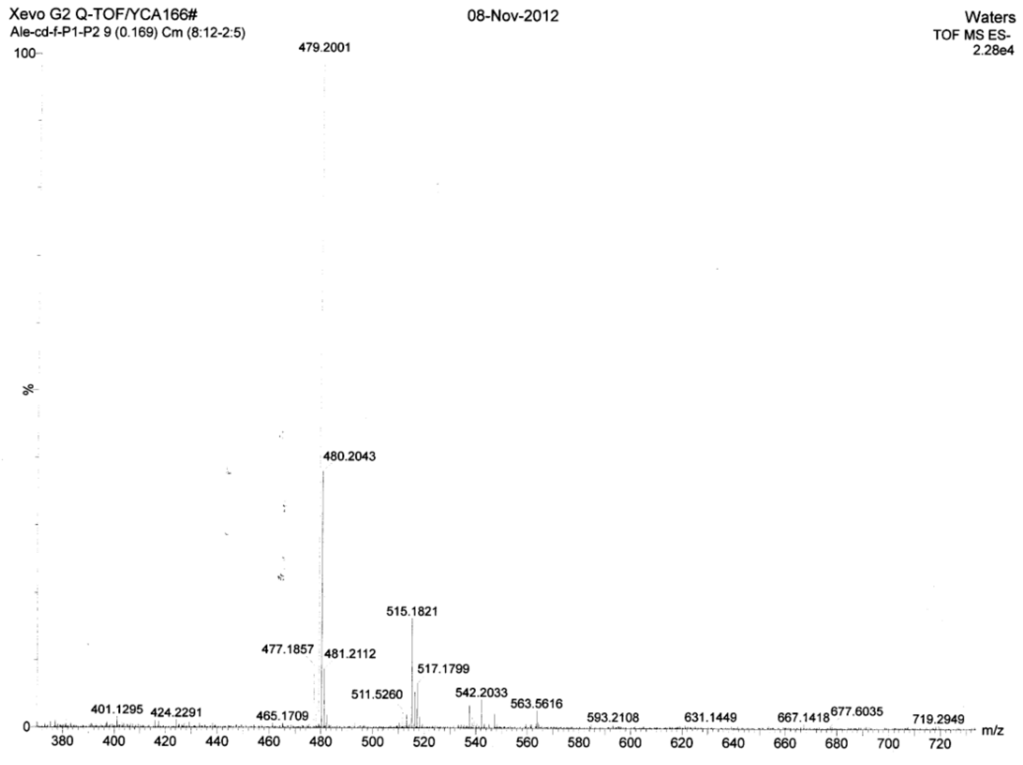

Figure S42. ESI-MS spectrum of compound **7**.
